# Blood-Brain Barrier Alterations and Edema Formation in Different Brain Mass Lesions

**DOI:** 10.3389/fncel.2022.922181

**Published:** 2022-07-15

**Authors:** Peter Solar, Michal Hendrych, Martin Barak, Hana Valekova, Marketa Hermanova, Radim Jancalek

**Affiliations:** ^1^Department of Neurosurgery, St. Anne’s University Hospital Brno, Faculty of Medicine, Masaryk University, Brno, Czechia; ^2^Department of Neurosurgery, St. Anne’s University Hospital, Brno, Czechia; ^3^First Department of Pathology, St. Anne’s University Hospital Brno, Faculty of Medicine, Masaryk University, Brno, Czechia; ^4^First Department of Pathology, St. Anne’s University Hospital, Brno, Czechia

**Keywords:** blood-brain barrier, glioblastoma multiforme, brain edema, brain metastasis, brain lymphoma, brain abscess

## Abstract

Differential diagnosis of brain lesion pathologies is complex, but it is nevertheless crucial for appropriate clinical management. Advanced imaging methods, including diffusion-weighted imaging and apparent diffusion coefficient, can help discriminate between brain mass lesions such as glioblastoma, brain metastasis, brain abscesses as well as brain lymphomas. These pathologies are characterized by blood-brain barrier alterations and have been extensively studied. However, the changes in the blood-brain barrier that are observed around brain pathologies and that contribute to the development of vasogenic brain edema are not well described. Some infiltrative brain pathologies such as glioblastoma are characterized by glioma cell infiltration in the brain tissue around the tumor mass and thus affect the nature of the vasogenic edema. Interestingly, a common feature of primary and secondary brain tumors or tumor-like brain lesions characterized by vasogenic brain edema is the formation of various molecules that lead to alterations of tight junctions and result in blood-brain barrier damage. The resulting vasogenic edema, especially blood-brain barrier disruption, can be visualized using advanced magnetic resonance imaging techniques, such as diffusion-weighted imaging and apparent diffusion coefficient. This review presents a comprehensive overview of blood-brain barrier changes contributing to the development of vasogenic brain edema around glioblastoma, brain metastases, lymphomas, and abscesses.

## Introduction

Brain edema, a pathologic state defined by excessive fluid accumulation within the brain parenchyma, can be divided into cytotoxic, ionic, and vasogenic types. Cytotoxic brain edema is characterized by swelling of the cells caused by the influx of osmolytes such as Na^+^ and Cl^–^ and fluids intracellularly. This type of brain edema is dominant following brain ischemia and paucity of ATP. Subsequently, ionic or osmotic edema develops as a result of fluids and osmolytes redistribution between the extravascular and intravascular spaces. These differences lead to the water transfer into the brain parenchyma across the blood-brain barrier (BBB). Vasogenic edema results from the breakdown of BBB and typically occurs in the brain parenchyma surrounding nearly all malignant central nervous system (CNS) tumors, as well as brain abscesses (Leinonen et al., 2018; White et al., 2019).

The BBB comprises interacting cells such as endothelial cells, pericytes, and astrocytes. These cells, along with neurons, microglia, vascular smooth muscle cells, the basement membrane, and the extracellular matrix, form part of the so-called neurovascular unit (Muoio et al., 2014; Gorelick et al., 2017). An intact BBB is essential for maintaining homeostasis of the CNS by separating the intravascular from the interstitial compartment of the brain (Abbott et al., 2006; Coureuil et al., 2017). Therefore, any alteration in the BBB results in vasogenic brain edema (Wahl et al., 1988), which can be detected by magnetic resonance imaging (MRI; Dalby et al., 2021). Studies so far have focused mainly on changes in the BBB in the enhancing brain lesions (Arvanitis et al., 2020; Rosińska and Gavard, 2021). These sites are characterized by a leaky BBB that allows passage of large molecules such as contrast agents used for MRI showing up as enhancement on T1 weighted images (Cramer and Larsson, 2014). However, little is known about the differences in the BBB in the perilesional edema beyond the margins of the T1 contrast-enhancement ring. The presence of water within brain tissue manifests as long T2 relaxation times that lead to increased T2 values and thus determine the presence or absence of brain edema (Obenaus and Ashwal, 2008). However, T2 values do not specify the compartment in which water accumulates. Therefore, advanced MRI parameters such as quantitative diffusion-weighted imaging (DWI) and apparent diffusion coefficient (ADC) are used to visualize water movement between brain compartments. Low ADC values reflect restricted water diffusion caused by cellular swelling and reduced extracellular space (Badaut et al., 2011). On the other hand, high ADC values are a consequence of increased water mobility through a disrupted BBB (Yang et al., 2017). The movement of water in the extracellular space is also affected by increased cellularity, which is seen as a decline in ADC values (Guzman et al., 2008; Klimas et al., 2013). In some cases, however, even advanced MRI techniques, such as DWI and ADC, fail to differentiate pathologies characterized by perilesional edema that require different therapeutic approaches (Suh et al., 2018). Thus, it is more appropriate to focus on finding other possible methods of ADC map analysis to differentiate these brain pathologies. For this purpose, it is necessary to understand how perilesional edema differs at the cellular and molecular level. Our review focuses on pathophysiological mechanisms responsible for BBB changes in common brain lesions, which are characteristically accompanied by perifocal edema such as GBM, brain metastases, lymphomas, and abscesses.

## Blood-Brain Barrier

The BBB is composed of non-fenestrated capillaries and strictly regulates the movement of molecules from the intravascular to the interstitial compartment of the brain (Kang et al., 2013). The main cellular components of BBB are endothelial cells (ECs), astrocytes, and pericytes (Ballabh et al., 2004). Endothelial cells line the vascular lumen and are connected at the molecular level by different junctional protein complexes such as tight junctions (TJ), adherent junctions (AJ), and gap junctions (Begley and Brightman, 2003; Solár et al., 2022). TJs are located between endothelial cells and consist of transmembrane proteins, including occludin, claudin, junctional adhesion molecule (JAM) as well as cytoplasmatic proteins that anchor transmembrane proteins to the cytoskeleton of endothelial cells (Stamatovic et al., 2016). The transmembrane protein occludin is considered important to maintaining BBB integrity. Moreover, occludin stretches the TJ and limits the transfer of small molecules through the BBB and thus participates in controlling endothelial paracellular permeability (Hashimoto and Campbell, 2020). Claudins, as other transmembrane proteins, belong to a group of more than 20 proteins that affect BBB permeability to molecules of a certain size and play a role in barrier formation (Hashimoto and Campbell, 2020). Claudins also contribute to high electrical resistance across the barrier by selectively limiting paracellular ion movement (Van Itallie and Anderson, 2004). The role of JAMs (mainly JAM-A but also JAM-B and JAM-C) is to maintain TJ permeability (Aurrand-Lions et al., 2001). Moreover, these TJ proteins regulate the level of integrin expression during pathological conditions and can thus affect leukocyte trafficking into the brain (Kummer and Ebnet, 2018). The transmembrane TJ proteins bind to the cell cytoskeleton through cytoplasmic proteins called zonulins [(ZO), ZO-1, ZO-2, and ZO-3] and cingulin, members of the membrane-associated guanylate kinase (MAGUK) family of proteins (Mitic et al., 2000; Hawkins and Davis, 2005).

Adherent junctions (AJ) are formed by transmembrane glycoproteins, cadherins, cytoplasmic proteins, p120, and catenins α, β, and γ. These proteins are located on the basolateral cell membrane. Cadherins interact with the cytoskeleton of endothelial cells through catenins and p120, members of the Armadillo protein family (Li et al., 2018). Vascular endothelial cadherin (VE-cadherin), a cadherin family protein, plays an important role during the morphogenesis of blood vessels as well as in maintaining cell-cell junction stability and BBB integrity (Vestweber, 2008; Li et al., 2018).

Gap junctions (GJ) are specialized membrane protein structures between adjacent endothelial cells that contribute to maintaining TJ integrity and allow direct intercellular communication (Sweeney et al., 2019). GJ comprise intercellular channels made up of transmembrane proteins called connexins, through which ions and small molecules can pass (Meşe et al., 2007; Stamatovic et al., 2016).

The basement membrane is a layer formed by a complex of extracellular matrix proteins and provides structural support for endothelial cells. The vascular basement membrane consists of a three-dimensional network and contains four major protein complexes made of laminins, collagen IV isoforms, heparan sulfate proteoglycans (perlecan), and nidogens. Collagen IV is the most abundant component of the basement membrane and interacts with endothelial cells and other basement membrane components (Xu et al., 2018). Laminins, a group of extracellular matrix glycoproteins, comprise three α, β, and γ chains and play a role in the organization of the basement membrane (Mak and Mei, 2017). Adhesion receptors play a vital role in maintaining BBB properties. These receptors, including integrins α1β1, α3β1, α6β1, αvβ1/αvβ3, and dystroglycan anchor cells to the basement membrane (Engelhardt and Sorokin, 2009). Integrins, as well as dystroglycan, connect the cytoskeleton of endothelial cells, pericytes, and astrocyte endfeet with extracellular matrix proteins (Del Zoppo et al., 2006; Engelhardt and Sorokin, 2009).

Pericytes are multifunctional cells with cytoplasmic processes attached to the basement membrane and surround the endothelial cells. Pericytes and endothelial cells are connected by different types of proteinaceous junctions such as TJ, GJ, and AJ (Allt and Lawrenson, 2001; Armulik et al., 2011). Moreover, synapse-like peg-socket contacts appear in places where endothelial cells and pericytes are in close contact. These specialized junctions are probably involved in the regulation of brain microcirculation. Other functions of pericytes include participating in the development of the BBB, influencing vascular permeability and inflammatory processes, as well as maintaining vessel stability through growth factors and angiogenic molecules (Dore-Duffy and LaManna, 2007; Nakagawa et al., 2007; Armulik et al., 2011).

Astrocytes are also an important component of the BBB. They are glial cells that interact with endothelial cells through their endfeet projections surrounding the abluminal side of capillaries. Astrocytes maintain homeostasis in the brain microenvironment, control neurotransmitter and ion concentrations, modulate synaptic transmission, and regulate immune reactions (Abbott et al., 2006; Keaney and Campbell, 2015).

## Glioblastoma

GBM IDH-wild-type is the most common primary tumor of the CNS in adults and is characterized by rapid diffuse infiltrative growth of neoplastic glial cells, predominantly with astrocytic differentiation (Ostrom et al., 2021). Tumor cells extend within the CNS mainly along white matter tracts, perivascular space, and subpial surface, which are detectable as secondary structures of diffusely growing GBM in histology samples (Zagzag et al., 2008). Generally, GBM is incurable with a dismal prognosis. Median overall survival is 16 months, notwithstanding standard treatment involving surgical resection followed by adjuvant concomitant chemo-radiotherapy with the alkylating agent temozolomide (Stupp et al., 2005; Lakomy et al., 2020; Tan et al., 2020). Even though GBM is one of the most vascularized neoplasms, antiangiogenic treatment-bevacizumab has exhibited limited effect in primary GBM and its use is currently limited for recurrent GBM (Tan et al., 2020).

### Vasculature of Glioblastoma

**FIGURE 1:**
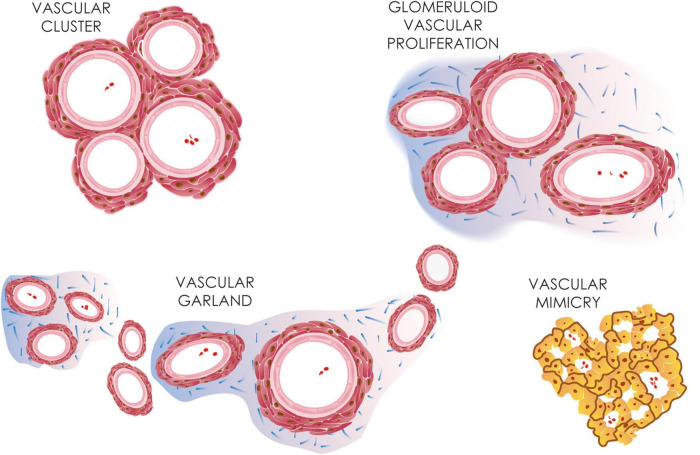
GBM microvasculature. Different microvasculature types detected in GBM, including vascular clusters, glomeruloid vascular proliferations, vascular garlands, and vascular mimicry. Vascular mimicry, unlike other microvascular types, shows a lumen lined by tumor cells.

Precipitative GBM growth necessitates a robust vascular system to supply the increasing nutrient and metabolic demands of the tumor mass. In contrast to the healthy brain vasculature with a highly organized physiological BBB, tumor vessels in GBM are remarkably irregular, structurally disorganized, and highly permeable, which results in irregular blood flow, vascular leakage, and brain edema. Morphologically GBM microvasculature might be divided into microvascular sprouting, which represents classic angiogenesis forming thin-walled capillaries, vascular mimicries, and bizarre vascular formations (Figure 1; Birner et al., 2003; Preusser et al., 2006; Chen et al., 2015).

**FIGURE 2:**
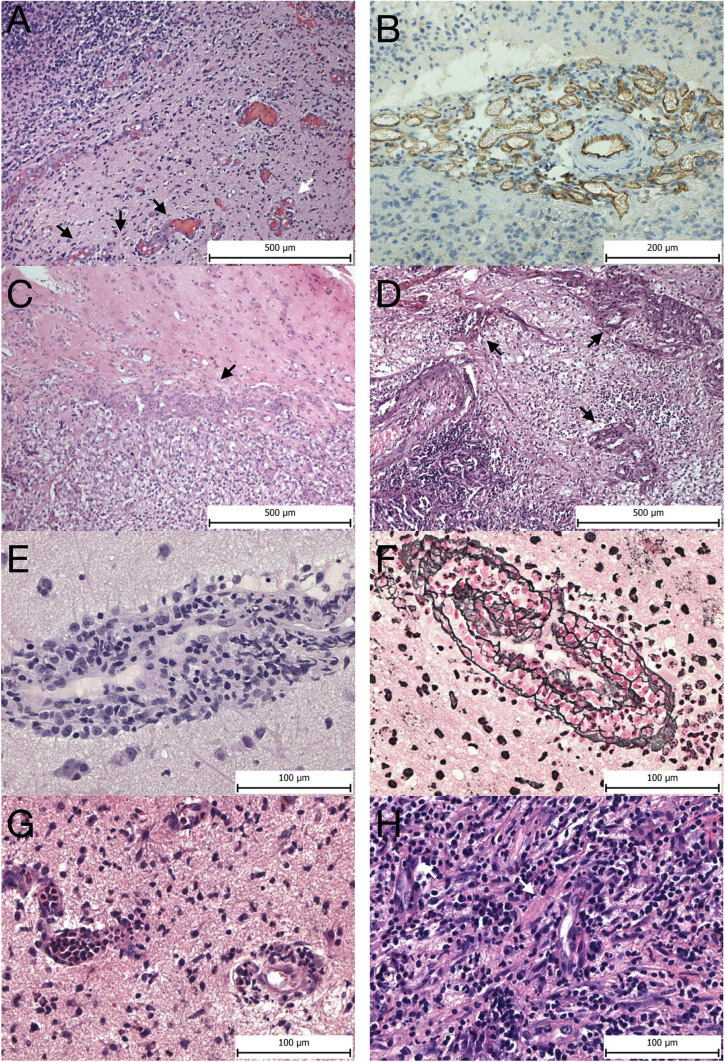
Vascular morphology. Microvascular proliferations at the invasive edge of GBM—**(A)** glomeruloid vascular proliferation (white arrow) and vascular garlands (black arrows). **(B)** CD31 expression in a vascular garland seen by immunohistochemistry. **(C)** Metastasis of clear cell renal cell carcinoma to the brain seen on the right side surrounded by microvascular proliferation (arrow). **(D)** Pulmonary non-mucinous adenocarcinoma metastatic to the brain (bottom left corner) surrounded by multiple glomeruloid microvascular proliferations (arrows). **(E)** Perivascular infiltration of the PCNSL intermingled with reactive lymphocytes forming vascular cuffs. **(F)** Reticulin-specific stain highlights the complex reticulin web encompassing the tumor cell caused by neoplastic lymphoid cells penetrating through the vascular wall. **(G)** Vessels in acute abscess in the brain with leukostasis and neutrophil transmigration across the BBB. H, vessels in chronic abscess display reactive pericytes prominently (white arrows), and no microvascular proliferations are seen. Panels **(A–D)** magnification 100x, panels **(E–H)** magnification 400x.

Vascular mimicry, also called vasculogenic mimicry, is the uncanny ability of tumor cells to assume endothelial-like properties, including expression of junction proteins such as VE-cadherin, and form a network of channels independent of endothelial cells reminiscent of a capillary system (Angara et al., 2017). The tumor cells are also surrounded by a rich extracellular matrix forming a basal membrane, which also contains laminins, proteoglycans, such as heparan sulfate as well as collagen IV and VI (Angara et al., 2017; Fernández-Cortés et al., 2019). Vascular mimicry is commonly found in aggressive malignant neoplasms and is linked with poorer prognosis, likely functionally contributing to tumor progression (Liu et al., 2011; Wang et al., 2013; Mei et al., 2020). Bizarre vascular formations, so-called microvascular proliferations (MPVs), are a histological hallmark of GBM. Nonetheless, their presence in the brain is not limited to GBM, as they have also been described in other primary or metastatic brain tumors. MVPs include vascular clusters, vascular garlands as well as glomeruloid vascular proliferations. Vascular clusters are local aggregations of more than three vessels lacking connective tissue ensheathing them (Figure 2). Vascular garlands are multiple vessels structured into complex spacious formations with garland-like architecture, and unlike glomeruloid vascular proliferations, they consist of tight vascular clusters encompassed by connective tissue. The presence of either vascular garlands or glomeruloid vascular proliferations is associated with worse disease outcomes in terms of progression-free survival (Chen et al., 2015). MPV density tends to increase from the tumor infiltrative zone toward the central perinecrotic zone in GBM (Takeuchi et al., 2010). MPVs are multilayered vessels formed of two distinct cell types. The inner luminar site is lined with flat endothelial cells enveloped by the smooth muscle cells/pericytes creating the outer abluminal surface, which is separated from the surrounding brain tissue infiltrated with GBM tumor cells by a network of reticulin/type III collagen fibers (Wesseling et al., 1995; Takeuchi et al., 2010). The disproportional cellular composition of MVPs with hypertrophic and hyperplastic pericytes is linked to their higher proliferation activity in relation to flattened endothelial cells (Díaz-Flores et al., 2021).

### Blood-Brain Barrier Disruption and Peritumoral Edema Formation in Glioblastoma

The neurovascular unit is a central component of the BBB that is dynamically modulated through tumor-neurovascular signaling. Glioma-induced BBB injury sequentially progresses to endothelial dysfunction followed by vasogenic edema. Subsequently, paracellular channels in the endothelium allow diffusion of plasmatic water molecules and proteins into the brain parenchyma, leading to characteristic peritumoral brain edema.

**FIGURE 3:**
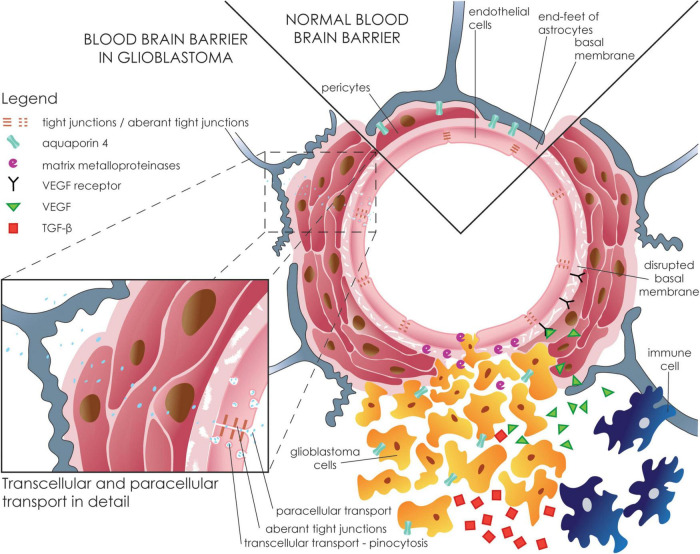
Disruption of the blood-brain barrier in glioblastoma. GBM cells infiltrate the perivascular space with subsequent astrocytic end-feet displacement. Tumor, stromal, and immune cells occupy a specific tumor niche with a diverse proteomic profile but most importantly show upregulated VEGF and TGF-ß. VEGF binds to its receptor on endothelial cells leading to increased transendothelial permeability and downregulation of specific TJs (e.g., claudin-5, occludin, and ZO-1) and subsequent increased paracellular influx. Matrix metalloproteinases, most importantly MMP-9, that are secreted by tumor cells contribute to the disruption of the basal membrane by cleaving the ECM. Inset shows a more detailed view of the transcellular and paracellular movements across the BBB. The endothelium forms the inner layer of the BBB. GBM promotes a “leaky” phenotype in endothelial cells with increased transcellular transport and endothelial fenestrations. Additionally, down-regulation of TJs leads to increased paracellular transport with subsequent edema formation.

The formation of vasogenic edema follows the structural disintegration of the endothelial BBB (Figure 3). Alteration in BBB has been shown to correspond to peritumoral edema in gliomas, suggesting a role for them in the formation of glioma-related edema. Downregulation of claudin-1, claudin-5, and occludin has been repeatedly reported in gliomas (Liebner et al., 2000; Karnati et al., 2014; Zhao et al., 2019). Moreover, the expression levels of claudin-1 and claudin-5 decrease with increasing severity of the glioma, according to World Health Organization (WHO) grading (Karnati et al., 2014). As for TJs expression, metalloproteinase-9 (MMP9) expression, which significantly contributes to GBM invasivity, leads to claudin-5 and occludin degradation (Xue et al., 2017; Zhao et al., 2019).

Similarly, down-regulation of claudin-1 and ZO-1 occurred through TGF-ß secretion, which is usually overexpressed in GBM (Ishihara et al., 2008; Han et al., 2015). However, the administration of anti-TGF-ß antibodies restored the barrier properties of the BBB (Ishihara et al., 2008). Up-regulation of claudin-5 and occludin is also seen upon glucocorticoid administration, a commonly used therapy to alleviate peritumoral edema in patients with GBM (Na et al., 2017). Interestingly, downregulation of plasma occludin levels has been shown to correlate with peritumoral brain edema volume and severity, suggesting that it may be used as a potential peritumoral edema biomarker (Park et al., 2006; Shi et al., 2020).

Several other mechanisms, including dysregulation in the expression of VEGF and aquaporins as well as pro-inflammatory changes, contribute to disruption in BBB and result in the development of vasogenic brain edema around GBM (Table 1).

**TABLE 1:** Table summarizing molecular and cellular changes in edematous brain tissue around glioblastoma, brain metastasis, lymphoma, and abscess.

	Molecular/Cellular target	Most relevant molecular results	References
Glioblastoma	Tight junctions	• Claudin-1 downregulation leads to altered TJ and increased endothelial permeability • Claudin-5 and occludin downregulated in hyperplastic tumor vessels	Liebner et al., 2000
		• Downregulation of claudin-1 and claudin-5 positively correlates with increasing glioma grade	Karnati et al., 2014
		• Decreased expression of BMP4 associated with downregulation of E-cadherin and claudin • Downregulation of BMP4 promotes tumor invasion	Zhao et al., 2019
		• Expression of MMP-9 positively correlates with increasing glioma grade • Overexpression of MMP-9 promotes glioblastoma cell proliferation	Xue et al., 2017
		• TGF-β downregulates expression of claudin-1 and promotes endothelial permeability • anti-TGF-β antibody reverts the downregulation of claudin-1 • TGF-β induced permeability reduced by MMP inhibition	Ishihara et al., 2008
		• Dexamethasone promotes the expression of claudin-5 and occludin. • Dexamethasone suppresses the expression of MMP-9	Na et al., 2017
		• Occludin expression correlates to peritumoral brain edema volume	Park et al., 2006
		• Serum occludin levels associated with peritumoral brain edema severity	Shi et al., 2020
	VEGF	• VEGF-A downregulates claudin-5 and occludin expression • Recombinant claudin-5 inhibits VEGF-induced paracellular hyperpermeability	Argaw et al., 2009
		• VEGF expression is induced under hypoxic conditions • VEGF is produced in proximity to necrotic glioblastoma	Shweiki et al., 1992
		• VEGF expression is lower in the peritumoral brain zone compared to the tumor core • Bevacizumab (anti-VEGF antibody) decreases microvessels density and normalizes vascular structures in the peritumoral brain zone	Tamura et al., 2018
		• HIF-1α upregulates VEGF expression	Lin et al., 2004
		• X-ray radiation promotes VEGF mRNA expression in glioblastoma cell cultures	Steiner et al., 2004
		• pSTAT3-VEGF signaling pathway associated with peritumoral brain edema volume	Wang et al., 2014
		• GBM-secreted VEGF downregulates claudin-5 in a dose-dependent manner and increases endothelial permeability	Wen et al., 2017
		• HIF-1α mediated STAT3 phosphorylation promotes glioma stem cell self-renewal under hypoxic conditions	Almiron Bonnin et al., 2018
		• VEGF stimulates glioblastoma stem cell proliferation *via* VEGFR-2	Xu et al., 2013
		• VEGFR-1 promotes glioblastoma cell migration and ECM invasion. • anti-VEGFR-1 antibody inhibits ECM invasion	Atzori et al., 2017
		• VEGF promotes inter-endothelial gaps, fragmentation of the endothelium, and degenerative changes in the vascular basement membrane	Dobrogowska et al., 1998
		• VEGF mRNA expression positively correlates with increased capillary permeability	Machein et al., 1999
		• VEGF promotes vascular hyperpermeability by the formation of vesiculo-vacuolar organelles • antisense VEGF reduces tumor edema	Lin et al., 2008
		• STAT3 pathway is constitutively activated in GBM	Brantley et al., 2008
		• GBM overexpresses pSTAT3 • Overexpression of pSTAT3 is a negative prognostic factor	Lin et al., 2014
	AQP4	• AQP4-OAP expression promotes morphological alterations of glioma cells • Expression of AQP-4 tetramers decreases cellular invasiveness, cellular migration, and • MMP-9 activity in glioma cells	Simone et al., 2019
		• Peritumoral brain edema in gliomas is associated with a dysfunction of the glymphatic system	Toh and Siow, 2021
		• Glioma cells overexpress AQP4 on their surface	Warth et al., 2004
		• AQP4 expression increased in GBM • Argin and dystroglycan regulates AQP4 localization • Upregulation of MMP-3 and MMP-9 follows disruption of argin and dystroglycan complexes in GBM	Noell et al., 2012
		• AQP4 redistribution on glioma cells surface highest in grade I and grade IV glioma • AQP4 overexpression is higher in the tumor core than in the peritumoral infiltration zone • AQP4 expression positively correlates with increasing peritumoral brain edema	Warth et al., 2007
		• AQP4 k/o mice displayed disrupted TJs and aberrant astrocytic endfeet • AQP4 loss associated with hyperpermeable BBB	Zhou et al., 2008
		• AQP4 overexpressed in high-grade glioma • AQP4 overexpression positively correlates with increasing BBB opening	Saadoun et al., 2002b
		• AQP4 overexpression appears to be a reaction to VEGF-induced edema in glioma • AQP4 expression is not directly driven by VEGF	Yang et al., 2012
		• AQP4 expression is higher in peritumoral tissue compared to tumor tissue • AQP4 overexpression positively correlates with edema index and degree of peritumoral edema • AQP4 redistributed in glioma • AQP4 correlates with VEGF and HIF-1α expression	Mou et al., 2010
		• AQP4 expression upregulated upon VEGF administration	Rite et al., 2008
		• Downregulation of AQP4 in endothelial argin k/o mice	Rauch et al., 2011
	Immune changes	• IL-1β production in microglia and bone marrow-derived macrophages blocked by dexamethasone • Dexamethasone reduces cytokine production in bone marrow-derived macrophages • Dexamethasone reduces TAM infiltration • Inhibition of the IL-1 signaling pathway decreases edema formation and reduces BBB permeability	Herting et al., 2019
		• Immune micro-environment in GBM predetermined by genetic driver mutations TAMs demonstrate heterogenous M0-, M1- and M2-like phenotypes	Chen et al., 2020
		• Microglia/macrophages infiltrate tumor tissue and regulate tumor invasion • TAMs do not produce pro-inflammatory cytokines • Reduction of immune cell pool attenuates tumor growth	Gabrusiewicz et al., 2011
		• Sulfasalazin treatment modulates tumor micro-environment	Sehm et al., 2016
		• IL-6 upregulates VEGF expression • IL-6 augments VEGF promoter activity in GBM	Loeffler et al., 2005
		• IL-1 receptor antagonist reduces glioma-related edema	Shevtsov et al., 2015
Brain metastasis	Tight junctions	• VEGF/VEGFR-2 pathway is implied in developing peritumoral brain edema. • Apatinib (tyrosine kinase inhibitor targeting VEGFR-2) decreases peritumoral edema volume	Song et al., 2018
		• Overexpression of SUR1 contributes to TJ disruption with subsequent peritumoral brain edema in melanoma metastases • SUR1 inhibitor as effective as dexamethasone in antiedematous therapy • Overexpression of SUR1 downregulates ZO-1	Thompson et al., 2013
		• Occludin downregulation leads to endothelial TJ disruption and increased BBB permeability	Papadopoulos et al., 2001
	VEGF	• VEGF promotes downstream Ras-ERK pro-angiogenic activity *via*αvβ5	Hood et al., 2003
		• Low levels of αvβ6 correlates with increased metastatic infiltration	Berghoff et al., 2013
		• Density of tumor-infiltrating lymphocytes associated with the extent of peritumoral edema • VEGF expressed by both melanoma cells and tumor-infiltrating lymphocytes	Trembath et al., 2020
		• cGMP induces hyperpermeability of the BBB	Chi et al., 1999
		• VEGF induces hyperpermeability of venules and capillaries • anti-VEGF monoclonal antibodies reverted hyperpermeable state	Roberts and Palade, 1995
		• VEGF induces permeability and dilatation of cerebral arterioles *via* release of NO/cGMP	Mayhan, 1999
	AQP4	• Anti-PD-1 therapy in melanoma decreases peritumoral edema volume	Tran et al., 2019
		• AQP4 overexpressed in peritumoral brain edema	Zhao et al., 2015
		• Glymphatic dysfunction contributes to the development of peritumoral brain edema	Toh et al., 2021
	Immune changes	• Inflammatory cells infiltrate peritumoral brain edema	Utsuki et al., 2007
		• Glial cells promote metastatic growth *via* secretion of multiple factors	Fitzgerald et al., 2008
		• Density of tumor-infiltrating lymphocytes positively correlates with peritumoral brain edema volume	Berghoff et al., 2016
	Others	• nNOS expression increased in high-grade tumors and melanoma metastasis • eNOS may promote brain edema	Broholm et al., 2003
		• AQP4 upregulated in astrocytes in edematous zones • Expression of α-syntrophin positively correlates with AQP4 upregulation	Saadoun et al., 2003
		• SUR1 upregulation linked to the development of cerebral edema in stroke • Inhibition of SUR1 reduces brain edema	Simard et al., 2006
		• SUR1 inhibitor reduces BBB disruption and caspase-3 activity in SAH	Simard et al., 2009
		• Astrocytic endothelin-1 overexpression leads to increased cerebral water content and increased expression of AQP4 • Vasopressin V2 receptor antagonist reduces cerebral edema and downregulates AQP4 expression	Yeung et al., 2009
		• Endothelin B receptor-mediates eNOS activation increases MMP-9 activity with subsequent downregulation of TJ • Endothelin B receptor antagonist decreases vasogenic brain edema in status epilepticus	Kim et al., 2015
		• TNF-α induces ET-1/eNOS activity with subsequent BBB hyperpermeability and vasogenic edema in status epilepticus	Kim et al., 2013
		• Smaller peritumoral brain edema characterized by lower HIF-1α expression and lower microvascular density	Spanberger et al., 2013
Brain lymphoma	VEGF	• VEGF expression associated with high microvascular density and alterations of BBB • VEGF expression leads to occludin and ZO-1 downregulation • VEGF expression associated with longer survival	Takeuchi et al., 2007
		• VEGF overexpression in Non-Hodgkin lymphoma outside the CNS is associated with a worse prognosis	Yang et al., 2015
	Endothelium	• Overexpression of pStat3 in PCNSL • Tumor cells may form tumor-associated endothelial microvessels	Ruggieri et al., 2017
		• Endothelial cells lack direct contact with astrocytic end-feet • Pericytes show irregular and discontinued basal membranes	Molnár et al., 1999
	Others	• Higher vascular permeability in PCNSL compared to glioblastoma as detected by MRI	Kickingereder et al., 2014
		• B-lymphoma cells infiltrate fiber tracks and in subarachnoid space • Reactive gliosis present in tumor tissue	Soussain et al., 2007
Brain abscess	VEGF	• VEGF upregulated in infiltrating inflammatory cells and perilesional astrocytes • VEGF contributes to the development of perilesional edema	Vaquero et al., 2001
	AQP4	• AQP4 has a protective role in the formation of peri-abscess edema in mouse abscess model • AQP4 promotes fluid reabsorption from the brain tissue around the abscess	Bloch et al., 2005
		• AQP4 downregulated in mouse brain abscess model • Nuclear factor erythroid-2 related factor depletion correlates with AQP4 downregulation in mouse brain abscess model	Ampawong and Luplertlop, 2019
	Immune changes	• TNF upregulation detrimental to brain edema • IL-10 k/o mouse brain abscess model leads to severe brain edema	Stenzel et al., 2005a
		• Brain edema significantly increased in TNF k/o mouse brain abscess model • TNF regulates immune response and controls edema formation in brain abscess	Stenzel et al., 2005b
		• PAMs-TLR-2 interaction promotes pro-inflammatory astrocytic activation	Esen et al., 2004
		• TLR2 promotes pro-inflammatory microglial activation	Kielian et al., 2005a
		• TLR2 regulates pro-inflammatory mediators’ expression	Kielian et al., 2005b
		• MyD88 promotes a protective inflammatory response in mouse brain abscess mode • MyD88 k/o exaggerates cerebral edema	Kielian et al., 2007
		• Increased levels of potassium, zinc, iron, and copper ions found in brain abscess • Inhibition of GABA and glutamate receptor promotes ROS formation	Dahlberg et al., 2015a
		• Th1 and Th17 lymphocytes regulate immune cell infiltration and release of inflammatory mediators	Holley and Kielian, 2012
		• Expression of inflammatory mediators positively correlates with increased BBB disruption • BBB disruption also positively correlates with increased neutrophil and macrophage/microglia infiltration	Baldwin and Kielian, 2004
		• Neuroinflammation promotes immune cell-mediated secretion of MMPs • MMPs degrade TJ and basement membrane	Rempe et al., 2016
		• Inflammatory response during cerebritis implied in BBB disruption and edema formation	Lo et al., 1994
		• CXC chemokines and MIP-2 that are expressed by resident glial cells implied in the recruitment of neutrophils	Kielian et al., 2001
	Others	• Elevated ammonia levels in brain abscess • Ammonia associated with neurotoxicity and brain edema	Dahlberg et al., 2016
		• Formation of ROS crucial in BBB disruption	Pun et al., 2009
		• GFAP k/o mice associated with poorly demarcated inflammatory lesions and severe brain edema • Astrocytic GFAP promotes restriction of pathogenic spread in brain abscess	Stenzel et al., 2004
		• Brain infection leads to increased levels of extracellular glutamate, GABA, and zinc	Hassel et al., 2014
		• Elevated extracellular levels of glutamate promote vascular hyperpermeability and BBB disruption through activation of NMDA receptors	Vazana et al., 2016

#### Vascular Endothelial Growth Factors and Blood-Brain Barrier Permeability in Glioblastoma

Vascular endothelial growth factors (VEGF) and their receptors (VEGFR) represent the main proangiogenic growth factors commonly involved in tumor neovascularization (Apte et al., 2019). Despite the proangiogenic properties of VEGF, it seems that overexpression of VEGF induces down-regulation of claudin-5 and occludin and thus potentiates the paracellular permeability of the BBB (Argaw et al., 2009).

Six VEGF isoforms have so far been identified, but it is the VEGF-A isoform that is most commonly implicated in the glioma-VEGF mediated signaling pathway (Greenberg and Jin, 2005). Needless to say, one of the intrinsic characteristics of glioma is VEGF overexpression (Shweiki et al., 1992; Tamura et al., 2018). VEGF overexpression has been shown to be driven by specific genetic mutations, by HIF-1α under hypoxic conditions, or through autocrine secretion in the tumor microenvironment (Lin et al., 2004; Steiner et al., 2004; Wang et al., 2014; Wen et al., 2017; Almiron Bonnin et al., 2018). Interestingly, VEGF mRNA expression corresponds to the WHO glioma grade, and VEGF-A distribution is specific, with the highest levels of VEGF in the hypoxic tumor core and lower values in the peritumoral zone (Tamura et al., 2018; Ahir et al., 2020). By binding to its respective receptors in the adjacent endothelium and glioma stem cells, VEGF promotes the invasive and proliferative properties of the glioma, thus leading to a hyperpermeable endothelium (Xu et al., 2013; Atzori et al., 2017). This VEGF-induced hyperpermeable endothelium with plasmatic extravasation into the brain parenchyma is the most common reason for the formation of inter-endothelial gaps, endothelial fenestrations, or fragmentations, and alterations in the basement membrane (Dobrogowska et al., 1998). Indeed, VEGF mRNA expression has been shown to correlate with the degree of capillary permeability (Machein et al., 1999). The administration of axitinib, a VEGF/VEGFR inhibitor, leads to increased integrity of the BBB (Wen et al., 2017). Further, VEGF also induces endothelium secreted matrix metalloproteinases with the subsequent disintegration of the extracellular matrix (ECM) and the basal membrane, as was described above (Ahir et al., 2020).

Alternatively, VEGF can induce hyperpermeability by forming vesiculo-vacuolar organelles (VVO) and thus increasing endothelial transcellular transport (Lin et al., 2008). One of the ways GBM harnesses VEGF overexpression is aberrant phosphorylation of STAT3 commonly found in GBM (Brantley et al., 2008; Lin et al., 2014; Ou et al., 2021). Phosphorylated STAT3 (pSTAT3) then upregulates transcription from the VEGF promoter and thus VEGF overexpression (Wang et al., 2014). Phosphorylated STAT3 (pSTAT3) then regulates the VEGF from the VEGF promoter leading to VEGF overexpression (Wang et al., 2014).

#### The Role of Aquaporins in Glioblastoma Induced Brain Edema Formation

Aquaporin is an integral membranous protein that acts as a water channel across cellular membranes (Takata et al., 2004). Numerous isoforms have been recognized, but aquaporin 1 (AQP1) and aquaporin 4 (AQP4) are the most prominent isoforms in the CNS (Verkman et al., 2011). In particular, numerous studies addressed the possible role of AQP4 in glioma-related edema, as well as in the regulation of glioma cell proliferation, invasivity, cell migration, and the brain glymphatic system (Yang et al., 2011; Lan et al., 2017; Hubbard et al., 2018; Simone et al., 2019; Toh and Siow, 2021). AQP4 is usually expressed at the astrocytic end-feet of the gliovascular interface of the BBB (Warth et al., 2004; Noell et al., 2012). These specific microdomains of the neurovascular unit facilitate dynamic changes in cellular volume and contribute to the regulation of the brain glymphatic system. However, gliomas are associated with aberrant spatial expression of astrocytic AQP4 and AQP4 redistribution across the glioma cell surface (Warth et al., 2007). Additionally, AQP4 k/o mice formed aberrant TJs and displayed astrocytic end-feet swelling (Zhou et al., 2008). In parallel, inactivation of the AQP4-associated Na^+^ channel SUR1-TRPM4 protein complex reverted astrocytic end-feet swelling (Stokum et al., 2018). Taken together, AQP4 expression is essential for the maintenance of the gliovascular interface (Zhou et al., 2008). However, upregulation of AQP4 was also linked to brain edema formation and BBB breakdown (Saadoun et al., 2002a; Yang et al., 2012). Interestingly, this upregulation was specific to certain WHO glioma grades, with the highest expression levels in grade I and IV gliomas (Warth et al., 2007). This correlates with the increased extent of peritumoral edema and increasing peritumoral AQP4 expression (Mou et al., 2010). Correspondingly, overexpression of VEGF in gliomas induces upregulation of AQP4 mRNA expression (Rite et al., 2008). Similarly, alterations in dystroglycan and argin complexes by MMP-2, MMP-3, and MMP-9 lead to edema formation through redistribution and abnormally directed AQP4 mediated water flow (Rauch et al., 2011; Noell et al., 2012).

In-depth molecular studies of AQP4 in GBM suggest that the state of AQP4 aggregation into AQP4 orthogonal arrays of particles (AQP4-OAPs) and disintegration of AQP4 into tetramers may be involved in determining the fate of glioma cells (Noell et al., 2012; Wolburg et al., 2012; Simone et al., 2019). Generally, AQP4-OAPs favor apoptotic glioma cell fate, while AQP4 tetramers increase glioma cell invasiveness. Interestingly, even though glioma tissue shows upregulated AQP4 expression, the expression levels of AQP4-OAPs remain unchanged (Lan et al., 2017).

#### Inflammation and Peritumoral Blood-Brain Barrier Disruption in Glioblastoma

The role of the specific immune microenvironment in cancer pathogenesis has been extensively investigated in the past few years. These dynamic interactions between glioma tumor cells and immune cells in the tumor microenvironment were shown to influence glioma invasion, infiltration, angiogenesis, or peritumoral edema (Hambardzumyan et al., 2016; Herting et al., 2019; Buonfiglioli and Hambardzumyan, 2021). Apart from glioma tumor cells, tumor-associated macrophages (TAMs) is the dominant non-neoplastic cell type in GBM (Buonfiglioli and Hambardzumyan, 2021). They represent a supportive stromal cell type providing advantageous conditions for GBM potentiation. Historically, an anti-inflammatory M2-like phenotype of TAMs was considered more implicated in GBM pathogenesis. However, recent transcriptomic studies have demonstrated that TAMs in GBM are rather heterogeneous comprising M0-, M1- and M2-like phenotypes leading to the production of both inflammatory and anti-inflammatory signaling molecules (Chen et al., 2020). Interestingly, TAMs express VEGF, IL-1, MMPs, or AQP4, which are all implicated in the pathogenesis of glioma-related edema (Saadoun et al., 2002a; Carlson et al., 2007; Gabrusiewicz et al., 2011; Herting et al., 2019). The upregulation of interleukin-1ß by TAMs has been associated with the suppression of astrocytic sonic-hedgehog signaling. This suppression leads to the downregulation of TJs and increased BBB permeability (Hambardzumyan et al., 2016). Herting et al. (2019) demonstrated that coculture of bone marrow-derived macrophages and organotypic tumor slices stimulated the secretion of both IL-1α and IL-1ß, while coculture with microglia yielded the opposite outcome. Additionally, inhibition of the IL-1 signaling pathway by ablation of the IL-1 receptor reduced BBB permeability and decreased edema formation in a mouse model. Furthermore, dexamethasone administration showed a similar IL-1α and IL-1ß down-regulation (Herting et al., 2019). Correspondingly, upon administration of sulfasalazine, an inhibitor of IL-1ß, cerebral edema decreased in size in the rat glioma model (Sehm et al., 2016). Apart from this, amplified expression of IL-6 has been associated with upregulation of VEGF secretion by GBM cells (Loeffler et al., 2005). Finally, glioma-related edema decreased upon administration of recombinant IL-1 receptor antagonists (Shevtsov et al., 2015).

## Brain Metastasis

Brain metastases are tumors primarily originating outside the CNS and spreading into the brain mainly through the hematogenous route or by direct infiltration from adjacent anatomic structures. Among those with the highest propensity to metastasize into the CNS are lung, breast, and renal cell carcinomas, along with melanomas. Brain metastases form solitary or multiple well-circumscribed masses with collateral brain edema (Ostrom et al., 2021).

**FIGURE 4:**
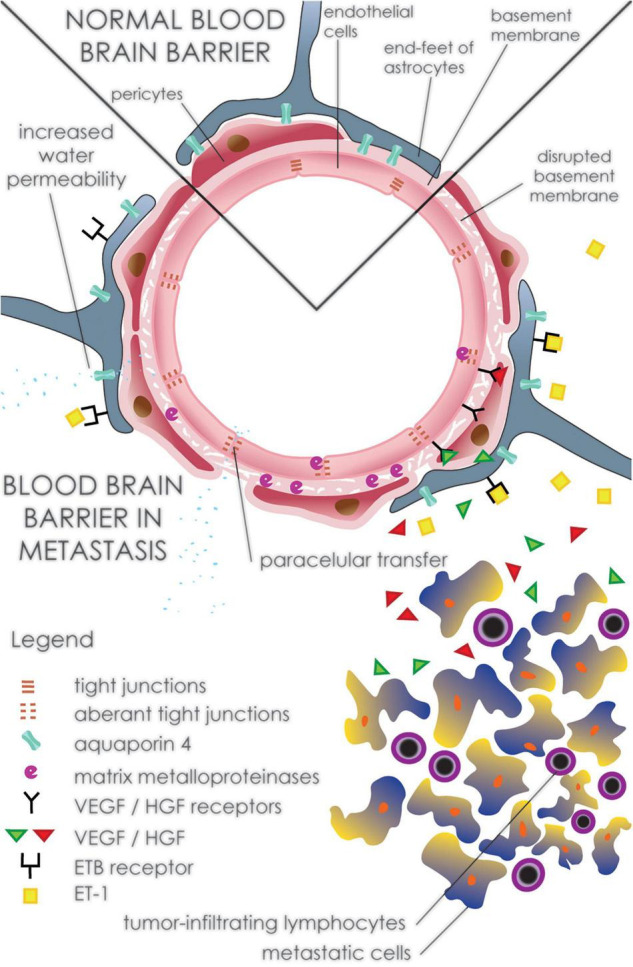
Disruption of the peritumoral blood-brain barrier in brain metastasis. Expression of VEGF and SH/VEGF by metastatic cells leads to alteration in TJ proteins such as occludin, claudin-1, and claudin-5 resulting in increased paracellular water influx. In addition to metastatic cells, tumor-infiltrating lymphocytes contribute to the production of VEGF as well as some cytokines including IL-2. Degradation of TJ proteins and the basal membrane is also potentiated by increased expression of matrix metalloproteinases such as MMP-2 and MMP-9. Activation of metalloproteinases is induced by overexpression of ET-1, which leads to up-regulation of ROS and eNOS. Increased expression of AQP4, a part of the glymphatic system on astrocytic endfeet around metastatic lesions, contributes to the development of vasogenic edema by the accumulation of CSF into the brain interstitium.

Disruption of BBB is considered one of the main causes of developing perilesional edema around brain metastases (Tran et al., 2019). Similar to GBM, decreased expression of the TJ protein occludin in brain metastasis contributes to the higher permeability of the BBB and the formation of peritumoral brain edema (Figure 4 and Table 1). Decreased expression of occludin was found not only in the tumor cells themselves but also in non-neoplastic endothelial cells adjacent to brain metastasis. As with GBM, VEGF plays an important role in these changes. Alterations in occludin expression may be caused by both upregulated VEGF and the secreted cytokine, Scatter Factor/Hepatocyte Growth Factor (SF/HGF). These secreted proteins are overexpressed by metastatic cells and may diffuse into peritumoral brain tissue (Papadopoulos et al., 2001). VEGF as well as SF/HGF induces the phosphorylation of occludin leading to its downregulation and subsequent opening of the BBB (Davies, 2002). Moreover, VEGF may induce fenestration in the endothelium and thus increase capillary permeability partially by inhibiting the TJ proteins claudin-1 and claudin-5 (Roberts and Palade, 1995; Song et al., 2018). Along with metastatic cells, tumor-infiltrating lymphocytes in the peritumoral edema also contribute to VEGF expression. This suggests that peritumoral edema is controlled not only by angiogenic cytokines from metastatic cells but also by infiltrating immune cells (Trembath et al., 2020).

VEGF action involves the synthesis and release of nitric oxide, activation of soluble guanylate cyclase and the consequent conversion of GTP to cGMP. This pathway of VEGF action contributes to BBB alteration and the development of peritumoral brain edema (Chi et al., 1999; Mayhan, 1999). The effect of NO on the development of edema around metastatic brain tumors can also be enhanced by increased expression of endothelial nitric oxide synthase (eNOS), not only in the tumor itself but also in the peritumoral brain area (Broholm et al., 2003).

VEGF has been found to cooperate with integrin αvβ5 and FAK and activate the Ras-ERK cascade. αvβ5 integrin expression was found on peritumoral vessels as well as on blood vessels in the surrounding brain parenchyma. On the other hand, increased expression of αvβ3 was found only on angiogenic, sprouting peritumoral vessels containing multilayered endothelial cells. Similarly, αvβ3, in collaboration with Basic Fibroblast Growth Factor (bFGF) receptor, activates downstream RAS and c-Raf, which leads to sustained Ras-ERK activation. These pathways regulate the expression of genes involved in cell survival, proliferation, differentiation, and migration (Hood et al., 2003; Berghoff et al., 2013). Moreover, it was shown experimentally that an increase in integrin αvβ3 leads to increased expression of MMP-2 and MMP-9, internalization of occludin and ZO-1, and the disruption of VE-cadherin localization (Edwards and Bix, 2019).

Activation of VEGF receptor 2 regulates the functions of vascular endothelium, leading to the generation of inositol trisphosphate (IP3) and diacylglycerol (DAG). These molecules increase the intracellular concentration of Ca^2+,^ which activates eNOS and, in turn, generates NO. The elevated level of intracellular calcium also stimulates the cytosolic phospholipase A (cPLA) and the production of prostaglandins. Vasoactive molecules, including NO and prostaglandins, lead to increased vascular cell permeability (Song et al., 2018). Other factors such as AQP4 and metalloproteinases are implicated in the development of brain peritumoral edema (Tran et al., 2019). This is supported by increased expression of AQP4 that was observed within peritumoral astrocytes adjacent to the brain metastasis (Saadoun et al., 2002b). Unlike peritumoral edema, AQP4 expression is decreased in the blood-tumor barrier (Zhao et al., 2015). In addition to AQP4, upregulation of Kir4.1 and α- syntrophin expression was found in astrocytes surrounding carcinoma metastasis (Saadoun et al., 2003). As part of the brain glymphatic system, increased expression of AQP4 channels in the peritumoral edema may be involved in elevated periarterial influx of CSF into the brain interstitium (Toh et al., 2021). Sulfonylurea receptor 1 (SUR1) may play a role in the development of cerebral metastatic-related vasogenic edema. Expression of SUR1 was found in glial and endothelial cells in the pericellular areas of the metastatic cells. Inhibition of SUR1 leads to decreased ZO-1 gap formation, suggesting that it acts on TJ proteins and thus on the BBB (Thompson et al., 2013). Moreover, SUR1 regulates the activity of the non-selective cation channel NCCa-ATP. Activation of these channels leads to Na^+^ influx followed by water influx to maintain osmotic neutrality resulting in cytotoxic edema. Elevated intracellular Na^+^ leads to alteration in the actin cytoskeleton and thus in the integrity of tight junctions. These changes increase paracellular plasma transfer and result in the development of vasogenic, plasma-rich edema (Simard et al., 2006, 2009).

Reactive glial cells participate in NO-independent pathways that may be involved in the development of peritumoral brain edema around brain metastases. Cells such as microglia and astrocytes were found to contribute to an inflammatory response in these areas (Utsuki et al., 2007; Fitzgerald et al., 2008). Reactive astrocytes produce endothelins that are released into the fluid microenvironment of the peritumoral brain (Zhang and Olsson, 1995). Endothelin-1 (ET-1), for instance, is known as a potent vasoconstrictor. In addition to the vasoconstrictive effect of ET-1, its overexpression in astrocytes could contribute to the development of cytotoxic edema (Yeung et al., 2009). ET-1 activates the ETB receptor, which subsequently induces the expression of eNOS, which in turn causes the activation of MMP-9 resulting in ZO-1 degradation and vasogenic edema formation (Kim et al., 2015). Moreover, activation of ETB receptors in astrocyte endfeet may generate intracellular reactive oxygen species (ROS) through the action of NADPH oxidase and cause dystrophin, the anchor protein for AQP4 to malfunction (Kim et al., 2013).

The microvascular density of brain metastases may influence the size of brain edema. High neovascularization and microvascular density were associated with large peritumoral edema (Figure 2). On the other hand, low microvascular density was found in brain metastases with small peritumoral edema. A similar pattern was found for hypoxia-induced factor 1 alpha (HIF1α) expression (Spanberger et al., 2013). The size of the peritumoral edema may reflect the extent of immune activation against brain metastases. In support of this, a correlation has been observed between the amount of peritumoral brain edema and the density of tumor-infiltrating CD8 + lymphocytes in brain metastases. The formation of edema is probably related to the release of cytokines such as IL-2 that stimulate T-lymphocytes (Berghoff et al., 2016).

Glymphatic pathways may also play a role in the development of peritumoral edema. Recently, it was suggested that brain metastases may disrupt movement in the glymphatic pathways. Altered glymphatic pathways complicate interstitial fluid clearance and contribute to its accumulation in the tumor interstitium and peritumoral edema formation (Toh et al., 2021).

## Brain Lymphoma

The CNS might be affected by a wide range of hematological neoplasms, either primarily originating within the CNS or by secondary spread from lymphomas that produce systemic disease. Primary CNS lymphomas (PCNSL) are a type of extranodal non-Hodgkin’s lymphoma, accounting for less than 1% of non-Hodgkin’s lymphomas. Of these PCNSLs, diffuse large cell B-cell lymphoma of the CNS is the most common histologically defined tumor (Wang et al., 2021). PCNSL typically forms either single or multiple hypercellular, diffusely growing lesions (Krebs et al., 2021). Predominant perivascular aggregation, in the form of tight vascular cuffs of neoplastic cells intermingled with reactive non-neoplastic infiltrating inflammatory lymphocytes and macrophages, is typically detected at the periphery (Marcelis et al., 2020). Nevertheless, PCNSLs are still poorly understood compared to GBM due to their significantly lower incidence.

**FIGURE 5:**
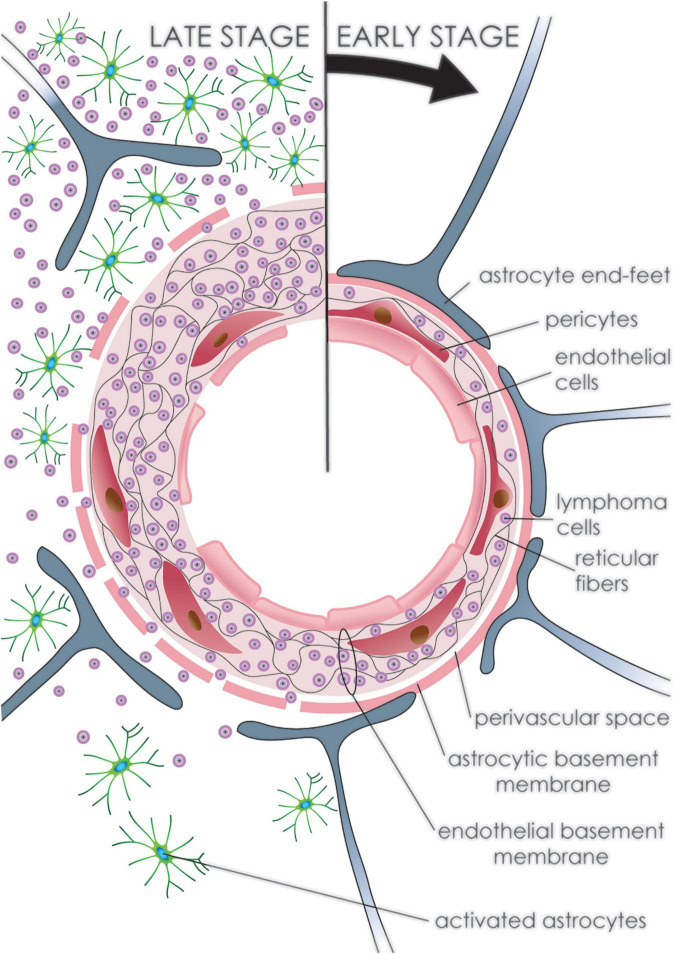
Development of primary CNS lymphoma. Progression of PCNSL (clockwise from top). Initially, neoplastic lymphoid cells accumulate within the vascular wall of arteries and venules fragmenting the reticular fiber network. The outer vascular layer with the glial end-feet maintains an impermeable barrier preventing tumor cells from infiltrating the brain tissue. Later, tumor cells fragment the outer layer and infiltrate brain tissue, where multiple reactive astrocytes can be detected. Endothelial cells undergo regressive as well as reparative changes throughout, leading to disruption of the endothelial lining.

A higher vascular permeability in PCNSL was detected by MRI imaging compared to GBM, which implies a greater degree of BBB disruption within the bulk tumor (Kickingereder et al., 2014). This BBB disruption within the tumor mass is seen as increased vessel permeability and corresponds to structural changes in microscopic observations. Vascular involvement is uneven, where neoplastic cells predominantly affect large vessels and not capillaries. Initially, the basal membrane and glial end-feet maintain an impermeable barrier, holding tumor cells within the vascular wall (Aho et al., 1993). Neoplastic PCNSL cells readily widen the co-opted vascular wall by penetrating through its rich reticulin fiber network, consisting of collagen type III and IV, laminin and fibronectin, thus forming a delicate web encompassing angiocentric tumor cells (Aho et al., 1993; Kickingereder et al., 2014). Later, the outer vascular reticulin layer in the PCNSL as well as in the secondary CNS lymphoma opens, which allows neoplastic lymphoid cells to infiltrate surrounding brain tissue (Figure 5 and Table 1). This brain tissue infiltration is rarely seen in reactive lymphocytic infiltration (Figure 2; Aho et al., 1993).

The endothelial cell lining, originating from vascular progenitor cells or presumably even from tumor cells, is frequently flattened with fenestrations, or is discontinuous, exposing a duplicated or frayed basal membrane (Ruggieri et al., 2017). Interestingly, a similar frequency of apoptotic endothelial cells was observed regardless of corticosteroid drug use. Intact endothelial cells display various degrees of regressive as well as regenerative changes. Endothelial cells frequently lack direct contact with normal astrocytic processes. Intercellular junctions between endothelial cells vary considerably in length, width, and density but appear to be functional. Pericytes are preserved in many cases and are intertwined in irregular and discontinued basal membranes (Molnár et al., 1999). These vascular changes are accompanied by reactive gliosis in the surrounding brain tissue (Soussain et al., 2007). Furthermore, significant variation has been detected in the preservation of BBB function in tumor-affected brain vessels in PCNSL showing varied VEGF expression. Tumors expressing VEGF presented with high microvascular density contain newly formed vessels and lack a well-formed BBB. Endothelial cells in immature PCNSL capillaries were fenestrated, similar to VEGF-induced hyperpermeable endothelium. Furthermore, the expression of occludin and ZO-1 was also significantly reduced. VEGF expressing PCNSL patients survived significantly longer when compared with the VEGF negative group, which exhibited preserved BBB function (Takeuchi et al., 2007). On the other hand, VEGF overexpression in non-Hodgkin’s lymphoma outside of the CNS is associated with a worse prognosis (Yang et al., 2015). Preserving BBB function in some PCNSL limits therapeutic agents from acting and thus might be directly linked with worse clinical outcomes. Furthermore, alterations in BBB function might occur to different degrees in various tumor lesions, of which some might remain completely unaltered and thus be virtually undetectable by current imaging while also being less available for therapeutic agents. This suggestion is supported clinically by frequent tumor relapse at a site distant from that of the initial tumor (Calimeri et al., 2021). Moreover, leaky BBB reconstitutes after therapy-induced tumor shrinkage and subsequent treatment is thus rendered less effective (Maza et al., 2009; Iorio-Morin et al., 2020). Autopsy studies have demonstrated that where imaging failed to detect the full extent of the tumor burden, PCNSL recurrence infiltrates the CNS diffusely (Lai et al., 2002).

## Brain Abscess

The most common causes of developing brain abscess are hematogenous spread, infected emboli, the extension of extracranial infections, penetrating head trauma, or complications of neurosurgical procedures. Nonetheless, in some cases, there is no obvious source. The most common organisms causing bacterial abscesses are *Staphylococcus aureus* and *Streptococcus* species (Mathisen and Johnson, 1997).

**FIGURE 6:**
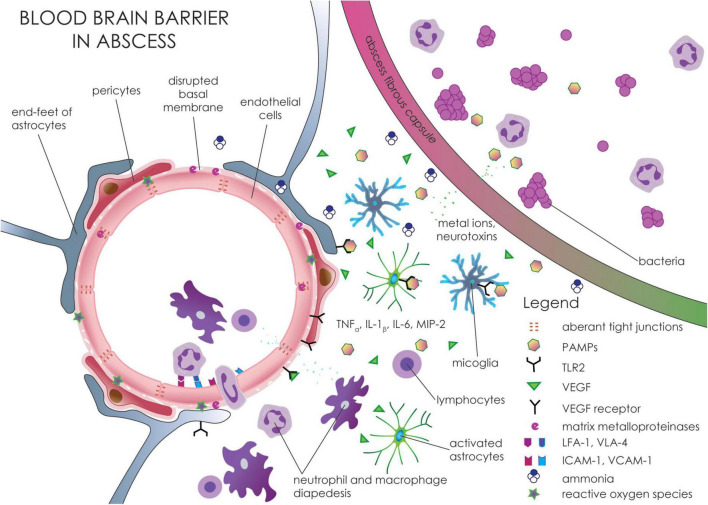
Molecular and cellular changes in the blood-brain barrier around brain abscess. Bacteria and other microorganisms in brain abscess release PAMPs that are recognized by TLR2 on astrocytes and microglia. Activation of TLR2 receptors leads to increased expression of various cytokines and chemokines, including TNF-α, IL-1β, IL-12, and MIP-2. These pro-inflammatory molecules increase the expression of adhesion molecules such as ICAM-1 and VCAM-1 that interact with integrins (LFA-1 and VLA-4) on immune cells and thus potentiate the transfer of leukocytes across the BBB. The pro-inflammatory molecules, as well as ROS, lead to increased expression and activation of matrix metalloproteinases resulting in disruption of TJ proteins. The formation of ROS is potentiated by metal ions, mainly potassium, zinc, and copper, that are released from the abscess. Moreover, decreased expression of TJ proteins such as claudin-5 and occludin is also potentiated by increased expression of VEGF from reactive astrocytes.

Disruption of the BBB following brain abscess leads to the development of vasogenic brain edema (Figure 6 and Table 1; Papadopoulos and Verkman, 2007). Changes in edematous brain tissue include shrunken cells with nuclear condensation resulting in pyknotic nuclei caused by neurotoxins such as α-hemolysin in *Staphylococcus* infection (Dahlberg et al., 2015b).

Cellular changes in the BBB include gaps in the endothelial cell layer (Wallenfang et al., 1980). Activated astrocytes form a diffusible barrier from fibrous tissue which surrounds the abscess. Some metal ions, mainly potassium and zinc, and others, including iron and copper, may penetrate from the abscess cavity into the brain tissue around the abscess. The elevated level of these ions in brain tissue leads to neuronal depolarization, inhibition of glutamate and GABA receptors, and formation of ROS that can contribute to BBB disruption (Pun et al., 2009; Hassel et al., 2014; Dahlberg et al., 2015a; Vazana et al., 2016). Moreover, higher concentrations of ammonia were found in brain edema. Elevated ammonia level in brain tissue around the abscess leads to swelling of astrocytes (Dahlberg et al., 2016). On the other hand, increased expression of the glial fibrillary acidic protein (GFAP) in activated astrocytes surrounding the brain abscess probably contributes to reducing the spread of these molecules and pathogens within the brain (Stenzel et al., 2004).

Reactive astrocytes and inflammatory cells are responsible for the production of VEGF, which affects BBB integrity in brain tissue surrounding the abscess (Vaquero et al., 2001). Upregulation of VEGF in reactive astrocytes leads to downregulation of claudin-5 and occludin, resulting in BBB disruption and the development of brain edema (Archie et al., 2021).

Accumulation of extracellular fluid following BBB disruption is reduced by upregulation of AQP4 expression in reactive astrocytes. Increased AQP4 expression was found in the capsule surrounding the abscess. AQP4 protection probably takes the form of fluid reabsorption from the brain tissue around the abscess and the consequent attenuation of vasogenic edema (Bloch et al., 2005). However, this effect may be attenuated by AQP4 downregulation in edematous brain vessels in the neighboring area of the brain (Ampawong and Luplertlop, 2019).

TNF-α plays an important role in the development of brain edema and the regulation of the immune response to brain abscesses (Stenzel et al., 2005a,b). Astrocytes and microglia are involved in the production of pro-inflammatory cytokines and chemokines that lead to BBB disruption and cause albumin and IgG accumulation in brain parenchyma resulting in the development of vasogenic edema. Pathogen-associated molecular patterns (PAMPs) released from bacteria are recognized by toll-like receptor 2 (TLR2) on astrocytes and microglia. Increased expression of TLR2 mediates glial activation in response to gram-positive bacteria such as *Staphylococcus aureus* (Esen et al., 2004; Kielian et al., 2005a). MyD88, the adapter protein for the majority of TLRs, is involved in downstream pathways and plays a role in the protective host CNS response during brain abscess development, thus also affecting the magnitude of brain edema (Kielian et al., 2007). Activation of TLR2 leads to increased expression of various cytokines such as TNF-α, IL-1β, IL-12, and chemokines including MIP-2, MIP-1α, β, MCP-1, and RANTES. It appears that TLR2 induces overexpression of pro-inflammatory molecules mainly during the acute stage of brain abscess (Kielian et al., 2005b). The released pro-inflammatory cytokines increase the expression of adhesion molecules, ICAM and VCAM that facilitate the movement of macrophages, neutrophils, and T cells across the BBB into the abscess (Figure 2; Kielian, 2004). Based on the observation that the fibrous capsule does not form a diffusion barrier (Dahlberg et al., 2015a), it is likely that these pro-inflammatory molecules can diffuse into the surrounding brain tissue and alter the BBB. CD3+, CD4+, Th1 and Th17 cells also seem to play an important role in the regulation of immune cell infiltration in the later stages of brain abscess. Th1 and Th17 regulate the release of inflammatory mediators from infiltrating macrophages and neutrophils (Holley and Kielian, 2012). Immune cells (mainly macrophages and neutrophils) release degradative enzymes such as metalloproteinases which lead to proteolysis of the basement membrane and TJ proteins and thus potentiate extravasation of peripheral immune cells through the BBB into the brain parenchyma (Baldwin and Kielian, 2004; Rempe et al., 2016). Rapid and sustained expression of CXC chemokines such as keratinocytes-derived chemokine (KC) and MIP-2 by resident glial cells play an important role in the recruitment of neutrophils (Kielian et al., 2001). BBB disruption and increased permeability in the early phase of brain abscess are important events in the inflammatory response and the development of abscess-associated brain edema (Lo et al., 1994).

## Summary

As described in this review, primary and secondary brain tumors or tumor-like brain lesions showed characteristic features of BBB damage resulting in the development of vasogenic brain edema. In general, BBB damage is mainly due to varying degrees of perilesional tumor cell infiltration, formation of new vessels, destruction of ECM, alteration of TJ proteins between endothelial cells, impaired cellular expression of growth factors, cellular channels, or inflammatory reactions. These changes are part of the complex pathophysiology of brain edema and present themselves to different extents in diagnoses like GBM, brain metastases, lymphomas, or abscesses. Future research should therefore focus on finding specific pattern(s) in vasogenic edema in different brain lesions that can be detected and quantified by advanced MRI techniques such as ADC maps. These patterns can then contribute to improved diagnosis so that appropriate treatment may be applied for these brain pathologies.

## Author Contributions

PS, MiH, and MB designed the study and wrote the manuscript. HV prepared the figures and participated in the preparation of the manuscript. MaH and RJ participated in the supervision and editing of the manuscript. All authors contributed to the article and approved the submitted version.

## Conflict of Interest

The authors declare that the research was conducted in the absence of any commercial or financial relationships that could be construed as a potential conflict of interest.

## Publisher’s Note

All claims expressed in this article are solely those of the authors and do not necessarily represent those of their affiliated organizations, or those of the publisher, the editors and the reviewers. Any product that may be evaluated in this article, or claim that may be made by its manufacturer, is not guaranteed or endorsed by the publisher.

## Abbreviations

ADC: apparent diffusion coefficient
CNS: central nervous system
GBM: glioblastoma
DWI: diffusion-weighted imaging
ADC: apparent diffusion coefficient
ECs: endothelial cells
TJ: tight junctions
AJ: adherent junctions
GJ: gap junctions
JAM: junctional adhesion molecule
ZO: zonula occludens
VE-cadherin: vascular endothelial cadherin
MPVs: microvascular proliferations
MMP: matrix metalloproteinase
VEGF: vascular endothelial growth factors
ECM: extracellular matrix
VVO: vesiculo-vacuolar organelles
pSTAT3: phosphorylated STAT3
AQP: aquaporin
AQP4-OAPs: orthogonal arrays of AQP4 particles
TAMs: tumor-associated macrophages
SF/HGF: scatter factor/hepatocyte growth factor
eNOS: endothelial nitric oxide synthase
bFGF: basic fibroblast growth factor
VEGFR2: vascular endothelial growth factor 2
IP3: inositol trisphosphate
DAG: diacylglycerol
cPLA: cytosolic phospholipase A
SUR1: sulfonylurea receptor 1
NCCa-ATP: ATP/Ca2 + non-selective cation channel
ET-1: endothelin-1
ROS: reactive oxygen species
HIF1 α: hypoxia-induced factor 1 alpha
PCNSL: primary CNS lymphomas
GFAP: glial fibrillary acidic protein
PAMPs: pathogen-associated molecular patterns
TLR2: toll-like receptor 2
KC: keratinocytes-derived chemokine.

## References

[B1] Abbott N. J. Rönnbäck L. Hansson E. (2006). Astrocyte-endothelial interactions at the blood-brain barrier. *Nat. Rev. Neurosci.* 7 41–53. 10.1038/nrn1824 16371949

[B2] Ahir B. K. Engelhard H. H. Lakka S. S. (2020). Tumor Development and Angiogenesis in Adult Brain Tumor: glioblastoma. *Mol. Neurobiol.* 57 2461–2478. 10.1007/s12035-020-01892-8 32152825 PMC7170819

[B3] Aho R. Ekfors T. Haltia M. Kalimo H. (1993). Pathogenesis of primary central nervous system lymphoma: invasion of malignant lymphoid cells into and within the brain parenchyme. *Acta Neuropathol.* 86 71–76. 10.1007/BF00454901 8372643

[B4] Allt G. Lawrenson J. G. (2001). Pericytes: cell biology and pathology. *Cells Tissues Organs* 169 1–11. 10.1159/000047855 11340256

[B5] Almiron Bonnin D. A. Havrda M. C. Lee M. C. Liu H. Zhang Z. Nguyen L. N. (2018). Secretion-mediated STAT3 activation promotes self-renewal of glioma stem-like cells during hypoxia. *Oncogene* 37 1107–1118. 10.1038/onc.2017.404 29155422 PMC5851110

[B6] Ampawong S. Luplertlop N. (2019). Experimental Scedosporiosis Induces Cerebral Oedema Associated with Abscess regarding Aquaporin-4 and Nrf-2 Depletions. *Biomed. Res. Int.* 2019:6076571. 10.1155/2019/6076571 31080825 PMC6475565

[B7] Angara K. Borin T. F. Arbab A. S. (2017). Vascular Mimicry: a Novel Neovascularization Mechanism Driving Anti-Angiogenic Therapy (AAT) Resistance in Glioblastoma. *Transl. Oncol.* 10 650–660. 10.1016/j.tranon.2017.04.007 28668763 PMC5496207

[B8] Apte R. S. Chen D. S. Ferrara N. (2019). VEGF in Signaling and Disease: beyond Discovery and Development. *Cell* 176 1248–1264. 10.1016/j.cell.2019.01.021 30849371 PMC6410740

[B9] Archie S. R. Al Shoyaib A. Cucullo L. (2021). Blood-Brain Barrier Dysfunction in CNS Disorders and Putative Therapeutic Targets: an Overview. *Pharmaceutics* 13:1779. 10.3390/pharmaceutics13111779 34834200 PMC8622070

[B10] Argaw A. T. Gurfein B. T. Zhang Y. Zameer A. John G. R. (2009). VEGF-mediated disruption of endothelial CLN-5 promotes blood-brain barrier breakdown. *Proc. Natl. Acad. Sci. U S A* 106 1977–1982. 10.1073/pnas.0808698106 19174516 PMC2644149

[B11] Armulik A. Genové G. Betsholtz C. (2011). Pericytes: developmental, physiological, and pathological perspectives, problems, and promises. *Dev. Cell* 21 193–215. 10.1016/j.devcel.2011.07.001 21839917

[B12] Arvanitis C. D. Ferraro G. B. Jain R. K. (2020). The blood-brain barrier and blood-tumour barrier in brain tumours and metastases. *Nat. Rev. Cancer* 20 26–41. 10.1038/s41568-019-0205-x 31601988 PMC8246629

[B13] Atzori M. G. Tentori L. Ruffini F. Ceci C. Lisi L. Bonanno E. (2017). The anti-vascular endothelial growth factor receptor-1 monoclonal antibody D16F7 inhibits invasiveness of human glioblastoma and glioblastoma stem cells. *J. Exp. Clin. Cancer Res.* 36:106. 10.1186/s13046-017-0577-2 28797294 PMC5553938

[B14] Aurrand-Lions M. Johnson-Leger C. Wong C. Du Pasquier L. Imhof B. A. (2001). Heterogeneity of endothelial junctions is reflected by differential expression and specific subcellular localization of the three JAM family members. *Blood* 98 3699–3707. 10.1182/blood.v98.13.3699 11739175

[B15] Badaut J. Ashwal S. Obenaus A. (2011). Aquaporins in cerebrovascular disease: a target for treatment of brain edema? *Cerebrovasc. Dis.* 31 521–531. 10.1159/000324328 21487216 PMC3085520

[B16] Baldwin A. C. Kielian T. (2004). Persistent immune activation associated with a mouse model of Staphylococcus aureus-induced experimental brain abscess. *J. Neuroimmunol.* 151 24–32. 10.1016/j.jneuroim.2004.02.002 15145600

[B17] Ballabh P. Braun A. Nedergaard M. (2004). The blood-brain barrier: an overview: structure, regulation, and clinical implications. *Neurobiol. Dis.* 16 1–13. 10.1016/j.nbd.2003.12.016 15207256

[B18] Begley D. J. Brightman M. W. (2003). ““Structural and functional aspects of the blood-brain barrier,”,” in *Peptide Transport and Delivery into the Central Nervous System Progress in Drug Research*, eds Prokai L. Prokai-Tatrai K. (Basel: Birkhäuser), 39–78. 10.1007/978-3-0348-8049-7_2 14674608

[B19] Berghoff A. S. Fuchs E. Ricken G. Mlecnik B. Bindea G. Spanberger T. (2016). Density of tumor-infiltrating lymphocytes correlates with extent of brain edema and overall survival time in patients with brain metastases. *Oncoimmunology* 5:e1057388. 10.1080/2162402X.2015.1057388 26942067 PMC4760339

[B20] Berghoff A. S. Rajky O. Winkler F. Bartsch R. Furtner J. Hainfellner J. A. (2013). Invasion patterns in brain metastases of solid cancers. *Neuro. Oncol.* 15 1664–1672. 10.1093/neuonc/not112 24084410 PMC3829586

[B21] Birner P. Piribauer M. Fischer I. Gatterbauer B. Marosi C. Ambros P. F. (2003). Vascular patterns in glioblastoma influence clinical outcome and associate with variable expression of angiogenic proteins: evidence for distinct angiogenic subtypes. *Brain Pathol.* 13 133–143. 10.1111/j.1750-3639.2003.tb00013.x 12744467 PMC8095831

[B22] Bloch O. Papadopoulos M. C. Manley G. T. Verkman A. S. (2005). Aquaporin-4 gene deletion in mice increases focal edema associated with staphylococcal brain abscess. *J. Neurochem.* 95 254–262. 10.1111/j.1471-4159.2005.03362.x 16181429

[B23] Brantley E. C. Nabors L. B. Gillespie G. Y. Choi Y.-H. Palmer C. A. Harrison K. (2008). Loss of PIAS3 Expression in Glioblastoma Multiforme Tumors: implications for STAT-3 Activation and Gene Expression. *Clin. Cancer Res.* 14 4694–4704. 10.1158/1078-0432.CCR-08-0618 18676737 PMC3886729

[B24] Broholm H. Rubin I. Kruse A. Braendstrup O. Schmidt K. Skriver E. B. (2003). Nitric oxide synthase expression and enzymatic activity in human brain tumors. *Clin. Neuropathol.* 22 273–281. 14672505

[B25] Buonfiglioli A. Hambardzumyan D. (2021). Macrophages and microglia: the cerberus of glioblastoma. *Acta Neuropathol. Commun.* 9:54. 10.1186/s40478-021-01156-z 33766119 PMC7992800

[B26] Calimeri T. Marcucci F. Corti A. (2021). Overcoming the blood-brain barrier in primary central nervous system lymphoma: a review on new strategies to solve an old problem. *Ann. Lymphoma* 5 10.21037/aol-20-54

[B27] Carlson M. R. J. Pope W. B. Horvath S. Braunstein J. G. Nghiemphu P. Tso C.-L. (2007). Relationship between survival and edema in malignant gliomas: role of vascular endothelial growth factor and neuronal pentraxin 2. *Clin. Cancer Res.* 13 2592–2598. 10.1158/1078-0432.CCR-06-2772 17473188

[B28] Chen L. Lin Z.-X. Lin G.-S. Zhou C.-F. Chen Y.-P. Wang X.-F. (2015). Classification of microvascular patterns via cluster analysis reveals their prognostic significance in glioblastoma. *Hum. Pathol.* 46 120–128. 10.1016/j.humpath.2014.10.002 25455996

[B29] Chen Z. Herting C. J. Ross J. L. Gabanic B. Puigdelloses Vallcorba M. Szulzewsky F. (2020). Genetic driver mutations introduced in identical cell-of-origin in murine glioblastoma reveal distinct immune landscapes but similar response to checkpoint blockade. *Glia* 68 2148–2166. 10.1002/glia.23883 32639068 PMC7512141

[B30] Chi O. Z. Liu X. Weiss H. R. (1999). Effects of cyclic GMP on microvascular permeability of the cerebral cortex. *Microvasc. Res.* 58 35–40. 10.1006/mvre.1999.2152 10388601

[B31] Coureuil M. Lécuyer H. Bourdoulous S. Nassif X. (2017). A journey into the brain: insight into how bacterial pathogens cross blood-brain barriers. *Nat. Rev. Microbiol.* 15 149–159. 10.1038/nrmicro.2016.178 28090076

[B32] Cramer S. P. Larsson H. B. (2014). Accurate Determination of Blood–Brain Barrier Permeability Using Dynamic Contrast-Enhanced T1-Weighted MRI: a Simulation and in vivo Study on Healthy Subjects and Multiple Sclerosis Patients. *J. Cereb. Blood Flow Metab.* 34 1655–1665. 10.1038/jcbfm.2014.126 25074746 PMC4269724

[B33] Dahlberg D. Ivanovic J. Hassel B. (2016). Toxic levels of ammonia in human brain abscess. *J. Neurosurg.* 124 854–860. 10.3171/2015.1.JNS142582 26274996

[B34] Dahlberg D. Mariussen E. Goverud I. L. Tønjum T. Mæhlen J. Antal E.-A. (2015b). Staphylococcal α-hemolysin is neurotoxic and causes lysis of brain cells in vivo and in vitro. *Neurotoxicology* 48 61–67. 10.1016/j.neuro.2015.03.001 25757835

[B35] Dahlberg D. Ivanovic J. Mariussen E. Hassel B. (2015a). High extracellular levels of potassium and trace metals in human brain abscess. *Neurochem. Int.* 82 28–32. 10.1016/j.neuint.2015.02.003 25684071

[B36] Dalby T. Wohl E. Dinsmore M. Unger Z. Chowdhury T. Venkatraghavan L. (2021). Pathophysiology of Cerebral Edema—A Comprehensive Review. *J. Neuroanaesth. Crit. Care* 08 163–172. 10.1055/s-0040-1721165

[B37] Davies D. C. (2002). Blood-brain barrier breakdown in septic encephalopathy and brain tumours. *J. Anat.* 200 639–646. 10.1046/j.1469-7580.2002.00065.x 12162731 PMC1570752

[B38] Del Zoppo G. J. Milner R. Mabuchi T. Hung S. Wang X. Koziol J. A. (2006). Vascular matrix adhesion and the blood-brain barrier. *Biochem. Soc. Trans.* 34 1261–1266. 10.1042/BST0341261 17073798

[B39] Díaz-Flores L. Gutiérrez R. González-Gómez M. García M.-D.-P. Díaz-Flores L. González-Marrero I. (2021). Disproportion in Pericyte/Endothelial Cell Proliferation and Mechanisms of Intussusceptive Angiogenesis Participate in Bizarre Vessel Formation in Glioblastoma. *Cells* 10:2625. 10.3390/cells10102625 34685606 PMC8534221

[B40] Dobrogowska D. H. Lossinsky A. S. Tarnawski M. Vorbrodt A. W. (1998). Increased blood-brain barrier permeability and endothelial abnormalities induced by vascular endothelial growth factor. *J. Neurocytol.* 27 163–173. 10.1023/a:1006907608230 10640176

[B41] Dore-Duffy P. LaManna J. C. (2007). Physiologic angiodynamics in the brain. *Antioxid. Redox Signal* 9 1363–1371. 10.1089/ars.2007.1713 17627476

[B42] Edwards D. N. Bix G. J. (2019). Roles of blood-brain barrier integrins and extracellular matrix in stroke. *Am. J. Physiol.-Cell Physiol.* 316 C252–C263. 10.1152/ajpcell.00151.2018 30462535 PMC6397343

[B43] Engelhardt B. Sorokin L. (2009). The blood-brain and the blood-cerebrospinal fluid barriers: function and dysfunction. *Semin. Immunopathol.* 31 497–511. 10.1007/s00281-009-0177-0 19779720

[B44] Esen N. Tanga F. Y. DeLeo J. A. Kielian T. (2004). Toll-like receptor 2 (TLR2) mediates astrocyte activation in response to the Gram-positive bacterium Staphylococcus aureus. *J. Neurochem.* 88 746–758. 10.1046/j.1471-4159.2003.02202.x 14720224

[B45] Fernández-Cortés M. Delgado-Bellido D. Oliver F. J. (2019). Vasculogenic Mimicry: become an Endothelial Cell “But Not So Much.”. *Front. Oncol.* 9:803. 10.3389/fonc.2019.00803 31508365 PMC6714586

[B46] Fitzgerald D. P. Palmieri D. Hua E. Hargrave E. Herring J. M. Qian Y. (2008). Reactive glia are recruited by highly proliferative brain metastases of breast cancer and promote tumor cell colonization. *Clin. Exp. Metastasis* 25 799–810. 10.1007/s10585-008-9193-z 18649117 PMC2679391

[B47] Gabrusiewicz K. Ellert-Miklaszewska A. Lipko M. Sielska M. Frankowska M. Kaminska B. (2011). Characteristics of the alternative phenotype of microglia/macrophages and its modulation in experimental gliomas. *PLoS One* 6:e23902. 10.1371/journal.pone.0023902 21901144 PMC3162015

[B48] Gorelick P. B. Furie K. L. Iadecola C. Smith E. E. Waddy S. P. Lloyd-Jones D. M. (2017). Defining Optimal Brain Health in Adults: a Presidential Advisory From the American Heart Association/American Stroke Association. *Stroke* 48:e284–e303. 10.1161/STR.0000000000000148 28883125 PMC5654545

[B49] Greenberg D. A. Jin K. (2005). From angiogenesis to neuropathology. *Nature* 438 954–959. 10.1038/nature04481 16355213

[B50] Guzman R. Altrichter S. El-Koussy M. Gralla J. Weis J. Barth A. (2008). Contribution of the apparent diffusion coefficient in perilesional edema for the assessment of brain tumors. *J. Neuroradiol.* 35 224–229. 10.1016/j.neurad.2008.02.003 18420272

[B51] Hambardzumyan D. Gutmann D. H. Kettenmann H. (2016). The role of microglia and macrophages in glioma maintenance and progression. *Nat. Neurosci.* 19 20–27. 10.1038/nn.4185 26713745 PMC4876023

[B52] Han J. Alvarez-Breckenridge C. A. Wang Q.-E. Yu J. (2015). TGF-β signaling and its targeting for glioma treatment. *Am. J. Cancer Res.* 5 945–955. 26045979 PMC4449428

[B53] Hashimoto Y. Campbell M. (2020). Tight junction modulation at the blood-brain barrier: current and future perspectives. *Biochim. Biophys. Acta Biomembr.* 1862:183298. 10.1016/j.bbamem.2020.183298 32353377

[B54] Hassel B. Dahlberg D. Mariussen E. Goverud I. L. Antal E.-A. Tønjum T. (2014). Brain infection with Staphylococcus aureus leads to high extracellular levels of glutamate, aspartate, γ-aminobutyric acid, and zinc. *J. Neurosci. Res.* 92 1792–1800. 10.1002/jnr.23444 25043715

[B55] Hawkins B. T. Davis T. P. (2005). The blood-brain barrier/neurovascular unit in health and disease. *Pharmacol. Rev.* 57 173–185. 10.1124/pr.57.2.4 15914466

[B56] Herting C. J. Chen Z. Maximov V. Duffy A. Szulzewsky F. Shayakhmetov D. M. (2019). Tumour-associated macrophage-derived interleukin-1 mediates glioblastoma-associated cerebral oedema. *Brain* 142 3834–3851. 10.1093/brain/awz331 31665239 PMC6906596

[B57] Holley M. M. Kielian T. (2012). Th1 and Th17 cells regulate innate immune responses and bacterial clearance during central nervous system infection. *J. Immunol.* 188 1360–1370. 10.4049/jimmunol.1101660 22190181 PMC3709259

[B58] Hood J. D. Frausto R. Kiosses W. B. Schwartz M. A. Cheresh D. A. (2003). Differential αv integrin–mediated Ras-ERK signaling during two pathways of angiogenesis. *J. Cell Biol.* 162 933–943. 10.1083/jcb.200304105 12952943 PMC2172815

[B59] Hubbard J. A. Szu J. I. Binder D. K. (2018). The role of aquaporin-4 in synaptic plasticity, memory and disease. *Brain Res. Bull.* 136 118–129. 10.1016/j.brainresbull.2017.02.011 28274814

[B60] Iorio-Morin C. Gahide G. Morin C. Vanderweyen D. Roy M.-A. St-Pierre I. (2020). Management of Primary Central Nervous System Lymphoma Using Intra-Arterial Chemotherapy With Osmotic Blood-Brain Barrier Disruption: retrospective Analysis of the Sherbrooke Cohort. *Front. Oncol.* 10:543648. 10.3389/fonc.2020.543648 33552946 PMC7855856

[B61] Ishihara H. Kubota H. Lindberg R. L. P. Leppert D. Gloor S. M. Errede M. (2008). Endothelial Cell Barrier Impairment Induced by Glioblastomas and Transforming Growth Factor β2 Involves Matrix Metalloproteinases and Tight Junction Proteins. *J. Neuropathol. Exp. Neurol.* 67 435–448. 10.1097/NEN.0b013e31816fd622 18431253

[B62] Kang E. J. Major S. Jorks D. Reiffurth C. Offenhauser N. Friedman A. (2013). Blood-brain barrier opening to large molecules does not imply blood-brain barrier opening to small ions. *Neurobiol. Dis.* 52 204–218. 10.1016/j.nbd.2012.12.007 23291193

[B63] Karnati H. K. Panigrahi M. Shaik N. A. Greig N. H. Bagadi S. A. R. Kamal M. (2014). Down Regulated Expression of Claudin-1 and Claudin-5 and Up Regulation of B-Catenin: association with Human Glioma Progression. *CNS Neurol. Disord. Drug Targets* 13 1413–1426. 10.2174/1871527313666141023121550 25345514 PMC6138250

[B64] Keaney J. Campbell M. (2015). The dynamic blood-brain barrier. *FEBS J.* 282 4067–4079. 10.1111/febs.13412 26277326

[B65] Kickingereder P. Sahm F. Wiestler B. Roethke M. Heiland S. Schlemmer H.-P. (2014). Evaluation of microvascular permeability with dynamic contrast-enhanced MRI for the differentiation of primary CNS lymphoma and glioblastoma: radiologic-pathologic correlation. *AJNR Am. J. Neuroradiol.* 35 1503–1508. 10.3174/ajnr.A3915 24722313 PMC7964431

[B66] Kielian T. (2004). Immunopathogenesis of brain abscess. *J. Neuroinflamm.* 1:16. 10.1186/1742-2094-1-16 15315708 PMC516022

[B67] Kielian T. Barry B. Hickey W. F. (2001). CXC chemokine receptor-2 ligands are required for neutrophil-mediated host defense in experimental brain abscesses. *J. Immunol.* 166 4634–4643. 10.4049/jimmunol.166.7.4634 11254722

[B68] Kielian T. Esen N. Bearden E. D. (2005a). Toll-like receptor 2 (TLR2) is pivotal for recognition of S. aureus peptidoglycan but not intact bacteria by microglia. *Glia* 49 567–576. 10.1002/glia.20144 15593098 PMC2394509

[B69] Kielian T. Haney A. Mayes P. M. Garg S. Esen N. (2005b). Toll-like receptor 2 modulates the pro-inflammatory milieu in Staphylococcus aureus-induced brain abscess. *Infect. Immun.* 73 7428–7435. 10.1128/IAI.73.11.7428-7435.2005 16239543 PMC1273898

[B70] Kielian T. Phulwani N. K. Esen N. Syed M. Md Haney A. C. McCastlain K. (2007). MyD88-Dependent Signals Are Essential for the Host Immune Response in Experimental Brain Abscess. *J. Immunol.* 178 4528–4537. 10.4049/jimmunol.178.7.4528 17372011 PMC2094730

[B71] Kim J.-E. Ryu H. J. Kang T.-C. (2013). Status Epilepticus Induces Vasogenic Edema via Tumor Necrosis Factor-α/ Endothelin-1-Mediated Two Different Pathways. *PLoS One* 8:e74458. 10.1371/journal.pone.0074458 24040253 PMC3764062

[B72] Kim J. Y. Ko A.-R. Hyun H.-W. Kang T.-C. (2015). ETB receptor-mediated MMP-9 activation induces vasogenic edema via ZO-1 protein degradation following status epilepticus. *Neuroscience* 304 355–367. 10.1016/j.neuroscience.2015.07.065 26232046

[B73] Klimas A. Drzazga Z. Kluczewska E. Hartel M. (2013). Regional ADC measurements during normal brain aging in the clinical range of b values: a DWI study. *Clin. Imaging* 37 637–644. 10.1016/j.clinimag.2013.01.013 23462734

[B74] Krebs S. Barasch J. G. Young R. J. Grommes C. Schöder H. (2021). Positron emission tomography and magnetic resonance imaging in primary central nervous system lymphoma-a narrative review. *Ann. Lymph.* 5:15. 10.21037/aol-20-52 34223561 PMC8248935

[B75] Kummer D. Ebnet K. (2018). Junctional Adhesion Molecules (JAMs): the JAM-Integrin Connection. *Cells* 7:E25. 10.3390/cells7040025 29587442 PMC5946102

[B76] Lai R. Rosenblum M. K. DeAngelis L. M. (2002). Primary CNS lymphoma: a whole-brain disease? *Neurology* 59 1557–1562. 10.1212/01.wnl.0000034256.20173.ea 12451197

[B77] Lakomy R. Kazda T. Selingerova I. Poprach A. Pospisil P. Belanova R. (2020). Real-World Evidence in Glioblastoma: stupp’s Regimen After a Decade. *Front. Oncol.* 10:840. 10.3389/fonc.2020.00840 32719739 PMC7348058

[B78] Lan Y.-L. Wang X. Lou J.-C. Ma X.-C. Zhang B. (2017). The potential roles of aquaporin 4 in malignant gliomas. *Oncotarget* 8 32345–32355. 10.18632/oncotarget.16017 28423683 PMC5458289

[B79] Leinonen V. Vanninen R. Rauramaa T. (2018). ““Chapter 4 - Raised intracranial pressure and brain edema,”,” in *Handbook of Clinical Neurology Neuropathology*, eds Kovacs G. G. Alafuzoff I. (Amsterdam: Elsevier), 25–37. 10.1016/B978-0-12-802395-2.00004-3 28987174

[B80] Li W. Chen Z. Chin I. Chen Z. Dai H. (2018). The Role of VE-cadherin in Blood-brain Barrier Integrity Under Central Nervous System Pathological Conditions. *Curr. Neuropharmacol.* 16 1375–1384. 10.2174/1570159X16666180222164809 29473514 PMC6251046

[B81] Liebner S. Fischmann A. Rascher G. Duffner F. Grote E. H. Kalbacher H. (2000). Claudin-1 and claudin-5 expression and tight junction morphology are altered in blood vessels of human glioblastoma multiforme. *Acta Neuropathol.* 100 323–331. 10.1007/s004010000180 10965803

[B82] Lin C. McGough R. Aswad B. Block J. A. Terek R. (2004). Hypoxia induces HIF-1alpha and VEGF expression in chondrosarcoma cells and chondrocytes. *J. Orthop. Res.* 22 1175–1181. 10.1016/j.orthres.2004.03.002 15475194

[B83] Lin G.-S. Chen Y.-P. Lin Z.-X. Wang X.-F. Zheng Z.-Q. Chen L. (2014). STAT3 serine 727 phosphorylation influences clinical outcome in glioblastoma. *Int. J. Clin. Exp. Pathol.* 7 3141–3149. 25031733 PMC4097241

[B84] Lin Z. X. Yang L. J. Huang Q. Lin J. H. Ren J. Chen Z. B. (2008). Inhibition of tumor-induced edema by antisense VEGF is mediated by suppressive vesiculo-vacuolar organelles (VVO) formation. *Cancer Sci.* 99 2540–2546. 10.1111/j.1349-7006.2008.00974.x 19032372 PMC11158782

[B85] Liu X. Zhang Q. Mu Y. Zhang X. Sai K. Pang J. C.-S. (2011). Clinical significance of vasculogenic mimicry in human gliomas. *J. Neurooncol.* 105 173–179. 10.1007/s11060-011-0578-5 21533525 PMC3198193

[B86] Lo W. D. Wolny A. Boesel C. (1994). Blood-brain barrier permeability in staphylococcal cerebritis and early brain abscess. *J. Neurosurg.* 80 897–905. 10.3171/jns.1994.80.5.0897 8169631

[B87] Loeffler S. Fayard B. Weis J. Weissenberger J. (2005). Interleukin-6 induces transcriptional activation of vascular endothelial growth factor (VEGF) in astrocytes in vivo and regulates VEGF promoter activity in glioblastoma cells via direct interaction between STAT3 and Sp1. *Int. J. Cancer* 115 202–213. 10.1002/ijc.20871 15688401

[B88] Machein M. R. Kullmer J. Fiebich B. L. Plate K. H. Warnke P. C. (1999). Vascular endothelial growth factor expression, vascular volume, and, capillary permeability in human brain tumors. *Neurosurgery* 44 732–740. 10.1097/00006123-199904000-00022 10201297

[B89] Mak K. M. Mei R. (2017). Basement Membrane Type IV Collagen and Laminin: an Overview of Their Biology and Value as Fibrosis Biomarkers of Liver Disease. *Anat. Rec.* 300 1371–1390. 10.1002/ar.23567 28187500

[B90] Marcelis L. Antoranz A. Delsupehe A.-M. Biesemans P. Ferreiro J. F. Debackere K. (2020). In-depth characterization of the tumor microenvironment in central nervous system lymphoma reveals implications for immune-checkpoint therapy. *Cancer Immunol. Immunother.* 69 1751–1766. 10.1007/s00262-020-02575-y 32335702 PMC11027603

[B91] Mathisen G. E. Johnson J. P. (1997). Brain abscess. *Clin. Infect. Dis.* 25 763–779. 10.1086/515541 9356788

[B92] Mayhan W. G. (1999). VEGF increases permeability of the blood-brain barrier via a nitric oxide synthase/cGMP-dependent pathway. *Am. J. Physiol.* 276 C1148–C1153. 10.1152/ajpcell.1999.276.5.C1148 10329964

[B93] Maza S. Kiewe P. Munz D. L. Korfel A. Hamm B. Jahnke K. (2009). First report on a prospective trial with yttrium-90-labeled ibritumomab tiuxetan (Zevalin) in primary CNS lymphoma. *Neuro. Oncol.* 11 423–429. 10.1215/15228517-2008-108 19060176 PMC2743222

[B94] Mei X. Chen Y.-S. Zhang Q.-P. Chen F.-R. Xi S.-Y. Long Y.-K. (2020). Association between glioblastoma cell-derived vessels and poor prognosis of the patients. *Cancer Commun.* 40 211–221. 10.1002/cac2.12026 32359215 PMC7238665

[B95] Meşe G. Richard G. White T. W. (2007). Gap junctions: basic structure and function. *J. Invest. Dermatol.* 127 2516–2524. 10.1038/sj.jid.5700770 17934503

[B96] Mitic L. L. Van Itallie C. M. Anderson J. M. (2000). Molecular physiology and pathophysiology of tight junctions I. Tight junction structure and function: lessons from mutant animals and proteins. *Am. J. Physiol. Gastrointest. Liver Physiol.* 279 G250–G254. 10.1152/ajpgi.2000.279.2.G250 10915631

[B97] Molnár P. P. O’Neill B. P. Scheithauer B. W. Groothuis D. R. (1999). The blood-brain barrier in primary CNS lymphomas: ultrastructural evidence of endothelial cell death. *Neuro. Oncol.* 1 89–100. 10.1093/neuonc/1.2.89 11550310 PMC1920754

[B98] Mou K. Chen M. Mao Q. Wang P. Ni R. Xia X. (2010). AQP-4 in peritumoral edematous tissue is correlated with the degree of glioma and with expression of VEGF and HIF-alpha. *J. Neurooncol.* 100 375–383. 10.1007/s11060-010-0205-x 20467785

[B99] Muoio V. Persson P. B. Sendeski M. M. (2014). The neurovascular unit - concept review. *Acta Physiol.* 210 790–798. 10.1111/apha.12250 24629161

[B100] Na W. Shin J. Y. Lee J. Y. Jeong S. Kim W.-S. Yune T. Y. (2017). Dexamethasone suppresses JMJD3 gene activation via a putative negative glucocorticoid response element and maintains integrity of tight junctions in brain microvascular endothelial cells. *J. Cereb. Blood Flow Metab.* 37 3695–3708. 10.1177/0271678X17701156 28338398 PMC5718327

[B101] Nakagawa S. Deli M. A. Nakao S. Honda M. Hayashi K. Nakaoke R. (2007). Pericytes from brain microvessels strengthen the barrier integrity in primary cultures of rat brain endothelial cells. *Cell Mol. Neurobiol.* 27 687–694. 10.1007/s10571-007-9195-4 17823866 PMC11517186

[B102] Noell S. Wolburg-Buchholz K. Mack A. F. Ritz R. Tatagiba M. Beschorner R. (2012). Dynamics of expression patterns of AQP4, dystroglycan, agrin and matrix metalloproteinases in human glioblastoma. *Cell Tissue Res.* 347 429–441. 10.1007/s00441-011-1321-4 22307776

[B103] Obenaus A. Ashwal S. (2008). Magnetic resonance imaging in cerebral ischemia: focus on neonates. *Neuropharmacology* 55 271–280. 10.1016/j.neuropharm.2008.06.010 18601935

[B104] Ostrom Q. T. Cioffi G. Waite K. Kruchko C. Barnholtz-Sloan J. S. (2021). CBTRUS Statistical Report: primary Brain and Other Central Nervous System Tumors Diagnosed in the United States in 2014-2018. *Neuro. Oncol.* 23 iii1–iii105. 10.1093/neuonc/noab200 34608945 PMC8491279

[B105] Ou A. Ott M. Fang D. Heimberger A. B. (2021). The Role and Therapeutic Targeting of JAK/STAT Signaling in Glioblastoma. *Cancers* 13:437. 10.3390/cancers13030437 33498872 PMC7865703

[B106] Papadopoulos M. C. Saadoun S. Woodrow C. J. Davies D. C. Costa-Martins P. Moss R. F. (2001). Occludin expression in microvessels of neoplastic and non-neoplastic human brain. *Neuropathol. Appl. Neurobiol.* 27 384–395. 10.1046/j.0305-1846.2001.00341.x 11679090

[B107] Papadopoulos M. C. Verkman A. S. (2007). Aquaporin-4 and brain edema. *Pediatr. Nephrol.* 22 778–784. 10.1007/s00467-006-0411-0 17347837 PMC6904420

[B108] Park M.-W. Kim C.-H. Cheong J.-H. Bak K.-H. Kim J.-M. Oh S.-J. (2006). Occludin Expression in Brain Tumors and its Relevance to Peritumoral Edema and Survival. *Cancer Res. Treat.* 38 139–143. 10.4143/crt.2006.38.3.139 19771274 PMC2741679

[B109] Preusser M. Heinzl H. Gelpi E. Schonegger K. Haberler C. Birner P. (2006). Histopathologic assessment of hot-spot microvessel density and vascular patterns in glioblastoma: poor observer agreement limits clinical utility as prognostic factors: a translational research project of the European Organization for Research and Treatment of Cancer Brain Tumor Group. *Cancer* 107 162–170. 10.1002/cncr.21973 16721804

[B110] Pun P. B. L. Lu J. Moochhala S. (2009). Involvement of ROS in BBB dysfunction. *Free Radic. Res.* 43 348–364. 10.1080/10715760902751902 19241241

[B111] Rauch S. M. Huen K. Miller M. C. Chaudry H. Lau M. Sanes J. R. (2011). Changes in Brain β-Amyloid Deposition and Aquaporin 4 Levels in Response to Altered Agrin Expression in Mice. *J. Neuropathol. Exp. Neurol.* 70 1124–1137. 10.1097/NEN.0b013e31823b0b12 22082664 PMC3223604

[B112] Rempe R. G. Hartz A. M. Bauer B. (2016). Matrix metalloproteinases in the brain and blood–brain barrier: versatile breakers and makers. *J. Cereb. Blood Flow Metab.* 36 1481–1507. 10.1177/0271678X16655551 27323783 PMC5012524

[B113] Rite I. Machado A. Cano J. Venero J. L. (2008). Intracerebral VEGF injection highly upregulates AQP4 mRNA and protein in the perivascular space and glia limitans externa. *Neurochem. Int.* 52 897–903. 10.1016/j.neuint.2007.10.004 18022290

[B114] Roberts W. G. Palade G. E. (1995). Increased microvascular permeability and endothelial fenestration induced by vascular endothelial growth factor. *J. Cell Sci.* 108 2369–2379. 10.1242/jcs.108.6.2369 7673356

[B115] Rosińska S. Gavard J. (2021). Tumor Vessels Fuel the Fire in Glioblastoma. *Int. J. Mol. Sci.* 22:6514. 10.3390/ijms22126514 34204510 PMC8235363

[B116] Ruggieri S. Tamma R. Resta N. Albano F. Coccaro N. Loconte D. (2017). Stat3-positive tumor cells contribute to vessels neoformation in primary central nervous system lymphoma. *Oncotarget* 8 31254–31269. 10.18632/oncotarget.16115 28415725 PMC5458205

[B117] Saadoun S. Papadopoulos M. C. Bell B. A. Krishna S. Davies D. C. (2002a). The aquaporin-4 water channel and brain tumour oedema. *J. Anat.* 200 523–534. 10.1046/j.1469-7580.2002.00047_16.x

[B118] Saadoun S. Papadopoulos M. C. Davies D. C. Krishna S. Bell B. A. (2002b). Aquaporin-4 expression is increased in oedematous human brain tumours. *J. Neurol. Neurosurg. Psychiatr.* 72 262–265. 10.1136/jnnp.72.2.262 11796780 PMC1737753

[B119] Saadoun S. Papadopoulos M. C. Krishna S. (2003). Water transport becomes uncoupled from K+ siphoning in brain contusion, bacterial meningitis, and brain tumours: immunohistochemical case review. *J. Clin. Pathol.* 56 972–975. 10.1136/jcp.56.12.972 14645363 PMC1770130

[B120] Sehm T. Fan Z. Ghoochani A. Rauh M. Engelhorn T. Minakaki G. (2016). Sulfasalazine impacts on ferroptotic cell death and alleviates the tumor microenvironment and glioma-induced brain edema. *Oncotarget* 7 36021–36033. 10.18632/oncotarget.8651 27074570 PMC5094980

[B121] Shevtsov M. A. Nikolaev B. P. Yakovleva L. Y. Dobrodumov A. V. Zhakhov A. V. Mikhrina A. L. (2015). Recombinant interleukin-1 receptor antagonist conjugated to superparamagnetic iron oxide nanoparticles for theranostic targeting of experimental glioblastoma. *Neoplasia* 17 32–42. 10.1016/j.neo.2014.11.001 25622897 PMC4309733

[B122] Shi S. Cheng J. Zhang C. Liang T. Zhang Y. Sun Y. (2020). Peripheral Blood Occludin Level as a Biomarker for Perioperative Cerebral Edema in Patients with Brain Tumors. *Dis. Markers* 2020 8813535. 10.1155/2020/8813535 32884584 PMC7455817

[B123] Shweiki D. Itin A. Soffer D. Keshet E. (1992). Vascular endothelial growth factor induced by hypoxia may mediate hypoxia-initiated angiogenesis. *Nature* 359 843–845. 10.1038/359843a0 1279431

[B124] Simard J. M. Chen M. Tarasov K. V. Bhatta S. Ivanova S. Melnitchenko L. (2006). Newly expressed SUR1-regulated NC(Ca-ATP) channel mediates cerebral edema after ischemic stroke. *Nat. Med.* 12 433–440. 10.1038/nm1390 16550187 PMC2740734

[B125] Simard J. M. Geng Z. Woo S. K. Ivanova S. Tosun C. Melnichenko L. (2009). Glibenclamide reduces inflammation, vasogenic edema, and caspase-3 activation after subarachnoid hemorrhage. *J. Cereb. Blood Flow Metab.* 29 317–330. 10.1038/jcbfm.2008.120 18854840 PMC2740919

[B126] Simone L. Pisani F. Mola M. G. De Bellis M. Merla G. Micale L. (2019). AQP4 Aggregation State Is a Determinant for Glioma Cell Fate. *Cancer Res.* 79 2182–2194. 10.1158/0008-5472.CAN-18-2015 30877104

[B127] Solár P. Zamani A. Lakatosová K. Joukal M. (2022). The blood–brain barrier and the neurovascular unit in subarachnoid hemorrhage: molecular events and potential treatments. *Fluids Barriers CNS* 19 1–79. 10.1186/s12987-022-00312-4 35410231 PMC8996682

[B128] Song Y. Liu B. Guan M. Liu M. (2018). Successful treatment using apatinib in intractable brain edema: a case report and literatures review. *Cancer Biol. Ther.* 19 1093–1096. 10.1080/15384047.2018.1491502 30081717 PMC6301820

[B129] Soussain C. Muldoon L. L. Varallyay C. Jahnke K. DePaula L. Neuwelt E. A. (2007). Characterization and magnetic resonance imaging of a rat model of human B-cell central nervous system lymphoma. *Clin. Cancer Res.* 13 2504–2511. 10.1158/1078-0432.CCR-06-2379 17438111

[B130] Spanberger T. Berghoff A. S. Dinhof C. Ilhan-Mutlu A. Magerle M. Hutterer M. (2013). Extent of peritumoral brain edema correlates with prognosis, tumoral growth pattern. *HIF1a expression and angiogenic activity in patients with single brain metastases*. *Clin. Exp. Metastas.* 30 357–368. 10.1007/s10585-012-9542-9 23076770

[B131] Stamatovic S. M. Johnson A. M. Keep R. F. Andjelkovic A. V. (2016). Junctional proteins of the blood-brain barrier: new insights into function and dysfunction. *Tissue Barriers* 4 e1154641. 10.1080/21688370.2016.1154641 27141427 PMC4836471

[B132] Steiner H.-H. Karcher S. Mueller M. M. Nalbantis E. Kunze S. Herold-Mende C. (2004). Autocrine pathways of the vascular endothelial growth factor (VEGF) in glioblastoma multiforme: clinical relevance of radiation-induced increase of VEGF levels. *J. Neurooncol.* 66 129–138. 10.1023/b:neon.0000013495.08168.8f 15015778

[B133] Stenzel W. Dahm J. Sanchez-Ruiz M. Miletic H. Hermann M. Courts C. (2005a). Regulation of the inflammatory response to Staphylococcus aureus-induced brain abscess by interleukin-10. *J. Neuropathol. Exp. Neurol.* 64 1046–1057. 10.1097/01.jnen.0000189836.48704.ca 16319715

[B134] Stenzel W. Soltek S. Miletic H. Hermann M. M. Körner H. Sedgwick J. D. (2005b). An essential role for tumor necrosis factor in the formation of experimental murine Staphylococcus aureus-induced brain abscess and clearance. *J. Neuropathol. Exp. Neurol.* 64 27–36. 10.1093/jnen/64.1.27 15715082

[B135] Stenzel W. Soltek S. Schlüter D. Deckert M. (2004). The intermediate filament GFAP is important for the control of experimental murine Staphylococcus aureus-induced brain abscess and Toxoplasma encephalitis. *J. Neuropathol. Exp. Neurol.* 63 631–640. 10.1093/jnen/63.6.631 15217091

[B136] Stokum J. A. Kwon M. S. Woo S. K. Tsymbalyuk O. Vennekens R. Gerzanich V. (2018). SUR1-TRPM4 and AQP4 form a heteromultimeric complex that amplifies ion/water osmotic coupling and drives astrocyte swelling. *Glia* 66 108–125. 10.1002/glia.23231 28906027 PMC5759053

[B137] Stupp R. Mason W. P. van den Bent M. J. Weller M. Fisher B. Taphoorn M. J. B. (2005). Radiotherapy plus Concomitant and Adjuvant Temozolomide for Glioblastoma. *N. Engl. J. Med.* 352 987–996. 10.1056/NEJMoa043330 15758009

[B138] Suh C. H. Kim H. S. Jung S. C. Kim S. J. (2018). Diffusion-Weighted Imaging and Diffusion Tensor Imaging for Differentiating High-Grade Glioma from Solitary Brain Metastasis: a Systematic Review and Meta-Analysis. *Am. J. Neuroradiol.* 39 1208–1214. 10.3174/ajnr.A5650 29724766 PMC7655439

[B139] Sweeney M. D. Zhao Z. Montagne A. Nelson A. R. Zlokovic B. V. (2019). Blood-Brain Barrier: from Physiology to Disease and Back. *Physiol. Rev.* 99 21–78. 10.1152/physrev.00050.2017 30280653 PMC6335099

[B140] Takata K. Matsuzaki T. Tajika Y. (2004). Aquaporins: water channel proteins of the cell membrane. *Prog. Histochem. Cytochem.* 39 1–83. 10.1016/j.proghi.2004.03.001 15242101

[B141] Takeuchi H. Hashimoto N. Kitai R. Kubota T. Kikuta K. (2010). Proliferation of vascular smooth muscle cells in glioblastoma multiforme. *J. Neurosurg.* 113 218–224. 10.3171/2009.10.JNS08631 19929197

[B142] Takeuchi H. Matsuda K. Kitai R. Sato K. Kubota T. (2007). Angiogenesis in primary central nervous system lymphoma (PCNSL). *J. Neurooncol.* 84 141–145. 10.1007/s11060-007-9363-x 17406788

[B143] Tamura R. Ohara K. Sasaki H. Morimoto Y. Yoshida K. Toda M. (2018). Histopathological vascular investigation of the peritumoral brain zone of glioblastomas. *J. Neurooncol.* 136 233–241. 10.1007/s11060-017-2648-9 29188530

[B144] Tan A. C. Ashley D. M. López G. Y. Malinzak M. Friedman H. S. Khasraw M. (2020). Management of glioblastoma: state of the art and future directions. *CA Cancer J. Clin.* 70 299–312. 10.3322/caac.21613 32478924

[B145] Thompson E. M. Pishko G. L. Muldoon L. L. Neuwelt E. A. (2013). Inhibition of SUR1 Decreases the Vascular Permeability of Cerebral Metastases. *Neoplasia* 15 535–543. 10.1593/neo.13164 23633925 PMC3638356

[B146] Toh C. H. Siow T. Y. (2021). Factors Associated With Dysfunction of Glymphatic System in Patients With Glioma. *Front. Oncol.* 11:744318. 10.3389/fonc.2021.744318 34631582 PMC8496738

[B147] Toh C. H. Siow T. Y. Castillo M. (2021). Peritumoral Brain Edema in Metastases May Be Related to Glymphatic Dysfunction. *Front. Oncol.* 11:4144. 10.3389/fonc.2021.725354 34722268 PMC8548359

[B148] Tran T. T. Mahajan A. Chiang V. L. Goldberg S. B. Nguyen D. X. Jilaveanu L. B. (2019). Perilesional edema in brain metastases: potential causes and implications for treatment with immune therapy. *J. Immunother. Cancer* 7:200. 10.1186/s40425-019-0684-z 31362777 PMC6668163

[B149] Trembath D. G. Davis E. S. Rao S. Bradler E. Saada A. F. Midkiff B. R. (2020). Brain Tumor Microenvironment and Angiogenesis in Melanoma Brain Metastases. *Front. Oncol.* 10:604213. 10.3389/fonc.2020.604213 33552976 PMC7860978

[B150] Utsuki S. Oka H. Sato S. Shimizu S. Suzuki S. Tanizaki Y. (2007). Histological examination of false positive tissue resection using 5-aminolevulinic acid-induced fluorescence guidance. *Neurol. Med. Chir.* 47 210–213. 10.2176/nmc.47.210 17527047

[B151] Van Itallie C. M. Anderson J. M. (2004). The role of claudins in determining paracellular charge selectivity. *Proc. Am. Thorac. Soc.* 1 38–41. 10.1513/pats.2306013 16113410

[B152] Vaquero J. Zurita M. Morales C. (2001). Possible Role for Vascular Permeability Factor in the Pathophysiology of Vasogenic Oedema Associated to Brain Abscess. *Acta Neurochir.* 143 1039–1040. 10.1007/s007010170009 11685611

[B153] Vazana U. Veksler R. Pell G. S. Prager O. Fassler M. Chassidim Y. (2016). Glutamate-Mediated Blood-Brain Barrier Opening: implications for Neuroprotection and Drug Delivery. *J. Neurosci.* 36 7727–7739. 10.1523/JNEUROSCI.0587-16.2016 27445149 PMC4951577

[B154] Verkman A. S. Ratelade J. Rossi A. Zhang H. Tradtrantip L. (2011). Aquaporin-4: orthogonal array assembly, CNS functions, and role in neuromyelitis optica. *Acta Pharmacol. Sin.* 32 702–710. 10.1038/aps.2011.27 21552296 PMC3601948

[B155] Vestweber D. (2008). VE-cadherin: the major endothelial adhesion molecule controlling cellular junctions and blood vessel formation. *Arterioscler. Thromb. Vasc. Biol.* 28 223–232. 10.1161/ATVBAHA.107.158014 18162609

[B156] Wahl M. Unterberg A. Baethmann A. Schilling L. (1988). Mediators of blood-brain barrier dysfunction and formation of vasogenic brain edema. *J. Cereb. Blood Flow Metab.* 8 621–634. 10.1038/jcbfm.1988.109 2843554

[B157] Wallenfang T. Bohl J. Kretzschmar K. (1980). Evolution of brain abscess in cats formation of capsule and resolution of brain edema. *Neurosurg. Rev.* 3 101–111. 10.1007/BF01644062 7231681

[B158] Wang H.-C. Hsiao H.-H. Du J.-S. Cho S.-F. Yeh T.-J. Gau Y.-C. (2021). Effect of Tumor Microenvironment and Angiogenesis on Clinical Outcomes of Primary Central Nervous System Lymphoma. *BioMed. Res. Int.* 2021:e3291762. 10.1155/2021/3291762 34631879 PMC8497102

[B159] Wang S.-Y. Ke Y.-Q. Lu G.-H. Song Z.-H. Yu L. Xiao S. (2013). Vasculogenic mimicry is a prognostic factor for postoperative survival in patients with glioblastoma. *J. Neurooncol.* 112 339–345. 10.1007/s11060-013-1077-7 23417321

[B160] Wang X.-F. Lin G.-S. Lin Z.-X. Chen Y.-P. Chen Y. Zhang J.-D. (2014). Association of pSTAT3-VEGF signaling pathway with peritumoral edema in newly diagnosed glioblastoma: an immunohistochemical study. *Int. J. Clin. Exp. Pathol.* 7 6133–6140. 25337261 PMC4203232

[B161] Warth A. Kröger S. Wolburg H. (2004). Redistribution of aquaporin-4 in human glioblastoma correlates with loss of agrin immunoreactivity from brain capillary basal laminae. *Acta Neuropathol.* 107 311–318. 10.1007/s00401-003-0812-0 14735305

[B162] Warth A. Simon P. Capper D. Goeppert B. Tabatabai G. Herzog H. (2007). Expression pattern of the water channel aquaporin-4 in human gliomas is associated with blood-brain barrier disturbance but not with patient survival. *J. Neurosci. Res.* 85 1336–1346. 10.1002/jnr.21224 17335082

[B163] Wen L. Tan Y. Dai S. Zhu Y. Meng T. Yang X. (2017). VEGF-mediated tight junctions pathological fenestration enhances doxorubicin-loaded glycolipid-like nanoparticles traversing BBB for glioblastoma-targeting therapy. *Drug Deliv.* 24 1843–1855. 10.1080/10717544.2017.1386731 29182025 PMC8241127

[B164] Wesseling P. Schlingemann R. O. Rietveld F. J. Link M. Burger P. C. Ruiter D. J. (1995). Early and extensive contribution of pericytes/vascular smooth muscle cells to microvascular proliferation in glioblastoma multiforme: an immuno-light and immuno-electron microscopic study. *J. Neuropathol. Exp. Neurol.* 54 304–310. 10.1097/00005072-199505000-00003 7745429

[B165] White M. L. Moore D. W. Zhang Y. Mark K. D. Greiner T. C. Bierman P. J. (2019). Primary central nervous system post-transplant lymphoproliferative disorders: the spectrum of imaging appearances and differential. *Insights Imaging* 10:46. 10.1186/s13244-019-0726-6 30972513 PMC6458224

[B166] Wolburg H. Noell S. Fallier-Becker P. Mack A. F. Wolburg-Buchholz K. (2012). The disturbed blood-brain barrier in human glioblastoma. *Mol. Aspects Med.* 33 579–589. 10.1016/j.mam.2012.02.003 22387049

[B167] Xu C. Wu X. Zhu J. (2013). VEGF promotes proliferation of human glioblastoma multiforme stem-like cells through VEGF receptor 2. *Sci. WorldJ.* 2013:417413. 10.1155/2013/417413 23533349 PMC3603324

[B168] Xu L. Nirwane A. Yao Y. (2018). Basement membrane and blood–brain barrier. *Stroke Vasc. Neurol.* 4 78–82. 10.1136/svn-2018-000198 31338215 PMC6613871

[B169] Xue Q. Cao L. Chen X.-Y. Zhao J. Gao L. Li S.-Z. (2017). High expression of MMP9 in glioma affects cell proliferation and is associated with patient survival rates. *Oncol. Lett.* 13 1325–1330. 10.3892/ol.2017.5567 28454256 PMC5403257

[B170] Yang J. Li Q. Wang Z. Qi C. Han X. Lan X. (2017). Multimodality MRI assessment of grey and white matter injury and blood-brain barrier disruption after intracerebral haemorrhage in mice. *Sci. Rep.* 7:40358. 10.1038/srep40358 28084426 PMC5234017

[B171] Yang J. Li W. He X. Zhang G. Yue L. Chai Y. (2015). VEGF Overexpression Is a Valuable Prognostic Factor for Non-Hodgkin’s Lymphoma Evidence from a Systemic Meta-Analysis. *Dis. Mark* 2015:786790. 10.1155/2015/786790 25810565 PMC4355555

[B172] Yang L. Wang X. Zhen S. Zhang S. Kang D. Lin Z. (2012). Aquaporin-4 upregulated expression in glioma tissue is a reaction to glioma-associated edema induced by vascular endothelial growth factor. *Oncol. Rep.* 28 1633–1638. 10.3892/or.2012.1973 22922737

[B173] Yang N. Ng Y. H. Pang Z. P. Südhof T. C. Wernig M. (2011). Induced neuronal cells: how to make and define a neuron. *Cell Stem Cell* 9 517–525. 10.1016/j.stem.2011.11.015 22136927 PMC4377331

[B174] Yeung P. K. K. Lo A. C. Y. Leung J. W. C. Chung S. S. M. Chung S. K. (2009). Targeted Overexpression of Endothelin-1 in Astrocytes Leads to More Severe Cytotoxic Brain Edema and Higher Mortality. *J. Cereb. Blood Flow Metab.* 29 1891–1902. 10.1038/jcbfm.2009.175 19707218

[B175] Zagzag D. Esencay M. Mendez O. Yee H. Smirnova I. Huang Y. (2008). Hypoxia- and vascular endothelial growth factor-induced stromal cell-derived factor-1alpha/CXCR4 expression in glioblastomas: one plausible explanation of Scherer’s structures. *Am. J. Pathol.* 173 545–560. 10.2353/ajpath.2008.071197 18599607 PMC2475791

[B176] Zhang M. Olsson Y. (1995). Reactions of astrocytes and microglial cells around hematogenous metastases of the human brain. Expression of endothelin-like immunoreactivity in reactive astrocytes and activation of microglial cells. *J. Neurol. Sci.* 134 26–32. 10.1016/0022-510x(95)00227-9 8747839

[B177] Zhao B. Wang H. Wang X. Zhao H. Liu J. (2015). Multiple intracranial metastatic tumor case report and aquaporin water channel-related research. *Cell Biochem. Biophys.* 71 1015–1021. 10.1007/s12013-014-0303-z 25342397

[B178] Zhao X. Sun Q. Dou C. Chen Q. Liu B. (2019). BMP4 inhibits glioblastoma invasion by promoting E-cadherin and claudin expression. *Front. Biosci.* 24:1060–1070. 10.2741/4768 30844730

[B179] Zhou J. Kong H. Hua X. Xiao M. Ding J. Hu G. (2008). Altered blood-brain barrier integrity in adult aquaporin-4 knockout mice. *Neuroreport* 19 1–5. 10.1097/WNR.0b013e3282f2b4eb 18281883

